# Anxiety, concerns and emotion regulation in individuals with Williams syndrome and Down syndrome during the COVID-19 outbreak: a global study

**DOI:** 10.1038/s41598-023-35176-7

**Published:** 2023-05-20

**Authors:** Vassilis Sideropoulos, Nayla Sokhn, Olympia Palikara, Jo Van Herwegen, Andrea C. Samson

**Affiliations:** 1 Department of Psychology and Human Development, IOE, UCL’s Faculty of Education and Society, 25 Woburn Square, London, UK; 2 Department of Psychology, University of Fribourg, Fribourg, Switzerland; 3 Eye and Brain Mapping Laboratory (iBMLab), Department of Psychology, University of Fribourg, Fribourg, Switzerland; 4 Department of Education Studies, University of Warwick, Coventry, UK; 5 Institute of Special Education, University of Fribourg, Fribourg, Switzerland; 6 Faculty of Psychology, UniDistance Suisse, Brig, Switzerland

**Keywords:** Emotion, Psychology, Human behaviour

## Abstract

Individuals with neurodevelopmental conditions (NDCs) have been reported to experience increased levels of anxiety during the COVID-19 pandemic. In our study, we document how individuals with Down Syndrome (DS; N = 557; Mage = 16.52; 233 female) and Williams syndrome (WS, N = 247; Mage = 18.43; 113 female) experienced the first wave (April 2020–May 2020) of the COVID-19 pandemic across the world. Using multilevel linear mixed regressions, we studied (a) parental reported anxiety of individuals with DS and WS, (b) these individuals’ specific concerns, and (c) their use and efficacy of emotion regulation (ER) strategies during the first wave of COVID-19. Predictors of anxiety, such as the age of the individual with NDC, type of condition, and time, were investigated. Individuals with WS experienced higher levels of anxiety compared to those with DS and the older the individuals with NDC were the more anxiety they experienced. In terms of concerns, group effects indicated that individuals with WS scored higher for most of the concerns. There were no gender differences in concerns, yet most of the concerns increased with age except for concerns about loss of routine, boredom, loss of institutional support and family conflict. Finally, significant group effects were found and indicated a more frequent use of a variety of adaptive and maladaptive ER strategies in individuals with WS. We did not identify group differences in the efficacy of ER strategies. Our results indicate that individuals with WS are likely to exhibit higher levels of anxiety, but also higher levels of concerns depending on their age. Similarly, individuals with WS use a variety of ER strategies more frequently but these strategies are not necessarily more efficient for them. We discuss the impact of these findings in relation to anxiety identification and support across individuals with NDCs.

## Introduction

Mental health conditions are on the rise amongst young people^1^, especially for those with neurodevelopmental conditions (NDCs)^2^ such as Williams Syndrome and Down Syndrome. Williams Syndrome (WS) is a genetic condition caused by microdeletion on the long arm of chromosome 7 and is characterized by moderate learning difficulties with average IQ scores of 50–60 and an uneven cognitive profile that includes weaker executive functioning, visuo-spatial abilities in contrast to their overall language abilities and strong short-term memory abilities^3^, although abilities are delayed compared to age-equivalent peers. Further to this, individuals with WS are more prone to several clinical and behavioural problems such as high levels of anxiety^4^, heart problems^5^ and difficulties with emotion regulation (ER) and maladaptive behaviours^6^. Down Syndrome (DS) is another genetic condition but is caused by trisomy or translocation of genetic materials on chromosome 21. Individuals with DS also have delayed cognitive development^7,8^ and overall IQ scores similar to those with WS, but have very different cognitive profiles with language development, especially speech production, being slow^9,10^; poor short-term memory^11^ and lower executive functioning^12^ compared to typically developing population. Individuals with DS have also complex clinical and behavioural profiles with several health problems such as thyroid disease^13^, high rates of psychopathology^14^ and maladaptive behaviours^15^. Nevertheless, not much is known about ER in DS. Whilst both individuals with WS and DS appear to have multifaceted cognitive, behavioural, and clinical profiles, they appear to have a very social nature and enjoy social activities^16,17^.

As a result of their cognitive, behavioural, and clinical profiles, many individuals with either WS or DS tend to fare better with structured days and routines^18,19^ and any uncertainty^20^ or disruption to their schedule is expected to affect them. The COVID-19 pandemic has created profound and novel shifts to our daily life, from moving to online-learning; working from home, and self-isolating to name a few. These day-to-day life disruptions have caused an increased global prevalence of anxiety and depression by 25% according to the World Health Organization^21^. When looking at the NDC population, previous research has highlighted how these disruptions have affected the wellbeing of individuals with NDC due to inability to access services, e.g., access to therapies^22,23^. Nevertheless, most studies focused on individuals with Autism or considered different individuals with different NDCs as one group but not separately. Therefore, understanding the implications of those changes upon individuals with WS and DS across the world is of great importance, considering the limited knowledge available in the literature of WS and DS on mental health.

### Mental health of individuals with WS and DS

Although mental health conditions are indeed on the rise for individuals with NDCs^1^, they are surprisingly less likely to be recognized, diagnosed, and treated^24,25^. Only a handful of previous studies have investigated mental health in WS and fewer have used standardized methods to assess mental health^26^. From those studies, it is reported that the rates of mental health difficulties in WS are considerably high, with anxiety being the main mental health issue^4,26^. The rates of anxiety conditions in WS are at 48%^27^ and individuals with WS often show specific anxieties, such as separation anxiety or anxiety related to health and hospitals. Furthermore, anxiety has been found to grow exponentially as they get older^26^. On the other hand, no gender differences for anxiety per se are reported in the literature of individuals with WS, apart from a study that argues that there is a female preponderance for depression, if the individual had a prior anxiety condition^28^.

Difficulties with ER are linked to problematic behaviour (maladaptive behaviours such as avoidance and withdrawal) and mental health issues (anxiety)^6^. Previous research suggests individuals with WS experience difficulties with ER in general^29^. Such limited control of emotions can lead to frustration and mood changes including anxiety^30^. While some evidence suggests that the ER problems are due to struggles with emotion processing in the WS population ^16^, other evidence argues that it is due to engagement with maladaptive behaviours. For example, withdrawal from social activities which in the long run can lead to isolation and anxiety. A meta-analysis suggests that despite the few studies examining maladaptive behaviours and ER in WS, the evidence indicates that the rates of problematic behaviour are high in that population, and individuals with WS engage more in maladaptive behaviours than in adaptive behaviour^31^. But further research is needed which should consider age, gender differences, and other socio-environmental factors^31^.

Despite the reporting that at least half of all individuals with DS will face a major mental health difficulty in their life^32^, the syndrome is associated with lower odds of mental health, specifically depression and anxiety when compared to other individuals with NDCs^14^ as well as to normative samples^33^. There is not enough evidence to infer whether factors such as age and gender play a key role for anxiety in individuals with DS as the current state of the literature is contradicting^15^. Nonetheless, the National Down Syndrome Society statistic puts those with DS at a similar risk to the general US population and complementary research argues that while they score lower for anxiety and depression, they still score higher for psychopathology^15^ and maladaptive behaviour^34^.

Such behaviour underlines the inability of individuals with DS to cope and regulate their emotions^35^ which can subsequently lead to higher levels of anxiety. The limited available evidence highlights that those with DS display high levels of emotion dysregulation^36,37^. Hence, they engage less in ER strategies which can explain the high levels of frustration reported in the DS population when compared to typically developing individuals^37^. However, it is very difficult to draw any concrete conclusions considering the gaps in the literature of DS when it comes to ER and the factors (age, gender) associated with it. Overall, individuals with DS appear to score lower for maladaptive behaviours compared to those with WS^38^, but further research is needed to unpick this complexity.

Taking all the evidence together, it is important to consider the diagnostic rates of mental health conditions in the NDC population. Studies on Autism report that autistic individuals are often misdiagnosed with mental health conditions due to lack of understanding autistic characteristics by health professionals^39^. Considering the above, and the lack of suitable assessments of mental health in WS and DS (no scale has been developed specifically for individuals with DS or WS), a similar case could be argued.

#### Mental health of individuals with WS and DS during COVID-19

Prior research has provided substantial evidence that emotional dysregulation^40^; lower executive functioning^12^ and higher intolerance of uncertainty^20^ affect the mental health of individuals with NDCs, including those with WS and DS. The emerging COVID-19 literature suggests an increase of anxiety and worries for individuals with NDCs compared to their typically developing siblings during the first lockdown in the UK and China^22,23,41^. Specifically, there was a surge in mood and behaviour changes during the COVID-19 pandemic. However, it is unclear whether all groups of NDCs are equally affected or what ER strategies they may have used to regulate emotions and the impact of anxiety caused by COVID-19.

For individuals with WS, there has been no independent or international research to examine the impact of the COVID-19 pandemic on anxiety or ER. However, preliminary findings state that individuals with WS experienced higher anxiety than other individuals with NDCs across the world as well as compared to pre-pandemic levels^42^.

Individuals with DS have been reported to be more irritable, angry, anxious, and depressed during the COVID-19 pandemic^43^. Similarly, they experienced increased rates of withdrawal during the first lockdown in Italy^44^. Yet, anxiety levels for DS population were lower when compared to individuals with other NDCs and their typically developing siblings in the UK^45^. Evidence also suggests that while individuals with DS exhibited lower anxiety, they still experienced elevated worries about how to approach others during the pandemic and how to engage with their friends^45^.

Researchers have raised concerns about the impact of COVID-19 on mental health due to the several restrictions imposed by different countries across the world and the series of lockdowns^46,47^. Evidence suggests that confinement and limited social interaction can lead to mood changes and can cause elevated rates of mental health difficulties, particularly for individuals with NDCs^48^. Similarly, research on ER strategies suggests that increased frequency of maladaptive behaviours was a key predictor of worsened mental health^49^. According to the literature, some maladaptive behaviours (e.g., isolation; withdrawal; information avoidance and information search) may be an unsuccessful attempt to attenuate anxiety levels, see^50^. Rumination and expressive suppression could possibly have a negative impact on emotions too^51,52^. In addition to the above, an increase on negative emotions and then anxiety levels can be caused by aggressive and repetitive behaviours^53,54^ while behaviours such as sharing/talking about a topic, distraction, cognitive reappraisal, focusing on the positive and humour may lead to increased positive and less negative emotions^52,55–57^. Overall, researchers have neglected the area of mental health (inclusive of ER) in populations such as WS and DS, which has great consequences as minimal knowledge exists on how to support these individuals in the recovery from COVID-19 and their re-integration to society such as going back school, enjoying activities, and engaging with friends. and engaging with friends.

Understanding the implications of these changes upon individuals with WS, DS and their parents/caregivers in an international context is thus important. The international approach allows for appropriate sample size for advanced statistical approaches and better insights on anxiety, concerns as well as ER strategies during uncertain times such as the COVID-19 pandemic.

### Current study

The aim of this paper was to examine the similarities and differences in how individuals with WS and DS have experienced the COVID-19 pandemic across the world, to allow for a large data sample, and to examine the impact of the pandemic on their mental health. We were interested in the anxiety over three time-points (time-point 1 = before the pandemic, time-point 2 = when the pandemic started, and time-point 3 = during the pandemic when the survey was completed), describing the main concerns at the time of completion of the parent-reported questionnaire with multi-level analyses with country as random factor and controlling for gender and age for individuals with WS and DS. We predicted that individuals with WS would exhibit higher levels of anxiety compared to individuals with DS. Controlling for gender is an important addition to the literature as previous research on mental health in typical populations highlights the differences between genders in terms of anxiety^58^. Further to this, to the best of our knowledge, there is no study to date which examined gender and age factors for mental health during the COVID-19 pandemic globally in individuals with NDCs. Due to the limited literature, no specific predictions were made.

Finally, we aimed to compare frequency and efficacy of 14 ER strategies between individuals with WS and DS at the time of completion of the survey in the early months of the COVID-19 pandemic using multilevel analyses controlling again for gender and age and using country as random factor. We focused on the most recent point as we wanted to capture engagement at that time and not recollective data considering this is the first international study on ER during the COVID-19 pandemic compared to other studies previously published on anxiety or depression (see below for detailed review). Our prediction was that those with WS will engage more frequently in maladaptive ER strategies than those with DS. However, individuals with DS will engage more efficiently in ER strategies that reduce their anxiety.

Overall, the current study provides an opportunity to understand the different elements that can affect anxiety of individuals with WS and DS during the COVID-19 and the ER strategies that could possibly promote or risk the individuals’ mental health. The decision to compare these two groups (WS and DS) was based on the similarities and differences between them: both have a high prevalence of medical issues (e.g., heart problems) but score differently on anxiety (higher anxiety in WS than DS).

## Method

### Procedure and study sample

Data for the current study were obtained by the Global Special Needs COVID-19 study which was available in 16 languages^59^. The data were collected between April and August of 2020. Parents and caregivers were asked to report about their child with NDCs. There was no age limit for the child with NDCs, so respondents were also able to report about their adult child. Data and scripts can be accessed through OSF: https://osf.io/5nkq9/.

For the present study, we compared 247 individuals with WS (*M* = 18.43 years, *SD* = 10.05, 113 female; Age range: 6–53) and 557 individuals with DS (*M* = 16.52 years, *SD* = 8.98, 233 female, Age range: 6 to 59) that have been selected based on the following criteria: (a) primary diagnosis of WS or DS, (b) available information about country, age, gender, available information about presence of intellectual disability (ID), (c) age 5 and older (since self-focused cognitive strategies are less likely to occur in younger participants). We excluded cases in which the respondents provided inconsistent information (e.g., primary diagnosis DS, but a negative response when asked if their child had ID). The individuals resided in 33 countries (see supplementary material A), specific data on race/ethnicity was not recorded. No gender differences were observed between the two groups; χ^2^ (1) = 0.92, *p* = 0.34. Mann–Whitney U test revealed a significant age difference between individuals with DS and WS: U = 61,713, *p* = *0.0*2.

Respondents were mainly mothers (71.52%), with 21.89% being fathers, and a minority (6.59%) were caregivers or other relatives. The education attainment levels of the respondents were 1.74% with no formal qualification, 14.59% with further vocational training, 21.32% with school leaving certificate, 31.92% with a university bachelor’s degree or equivalent, 25.31% with a university master’s degree or equivalent and 5.11% who wrote “Other”.

### Ethical approval

The Ethics Commission of UniDistance Suisse, Switzerland approved the anonymous online study. All respondents provided informed consent to participate in the online survey. All methods in the study were performed in accordance with the relevant guidelines and regulations of UniDistance Suisse as well as the Code of Ethics of the World Medical Association (Declaration of Helsinki).

### Measures

All measures and items used in the survey can be accessed freely on the OSF Website^59^: https://osf.io/5nkq9/. The anonymous online survey was divided into four segments where respondents had the opportunity to answer a range of open-ended and closed questions.

The first segment asked demographic questions about the respondents and the individual with NDCs, the primary diagnosis as well as the presence of any ID.

The second segment collected data on the anxiety level of the individual with NDCs. Anxiety was assessed on a single item which had a scale from 1 (not at all) to 5 (extremely) at three time-points: before the pandemic, at the start of the pandemic (these two time-points were retrospectively assessed) and in the current moment (between April and August 2020). The question we used was: “*How anxious was/is your child before/when it started/now?”.*

The third segment had questions about the concerns the individual experienced. Thirteen questions around concerns were asked as defined by previous researchers^60^ on top of additional items which were later grouped into the following categories: Health Related Concerns, Social Related Concerns, School Closure Related Concerns and Family Related Concerns. All concerns were rated on a 5-point Likert Scale; 1 (not at all) to 5 (extremely).

The last segment focused on the frequency of ER strategies and their efficacy. All items were rated again on a 5-point Likert Scale; for frequency: 1 (very rarely) to 5 (very frequently); and for efficacy: 1 (not efficient) to 5 (very efficient). In total there were 14 items, which focused on maladaptive and adaptive ER strategies and cognitive and behavioural strategies to capture the relative strengths of each group (see^61^ for examination of ER strategies use during COVID-19 for a range of neurodevelopmental populations including individuals with WS, but not for DS. Similarly that study did not examine efficacy of ER strategies which the present study furtherly explores). Whilst our measures were not standardised, recent research evidence argues that humans have the ability to successfully operationalise an integer scale for feelings, despite the fact that there is no such true scale^39^.

### Data analysis plan

Multiple linear mixed models (LMM) were computed to investigate the level of anxiety over three time-points (time-point 1 = before the pandemic, time-point 2 = when the pandemic started and time-point 3 = during the pandemic when the survey was completed) amongst the two groups (individuals with WS and DS). Interactions between time, age, gender, and group were also computed. Country and family identification code were used as a random factor, while we controlled for age and gender.

Next, 13 linear mixed models were computed to study the profiles of concerns in the two groups at time-point 3, where again we controlled for age and gender. Interactions between age, gender, and group were also computed. Country and family identification code were used as random factors while we controlled for age and gender.

Finally, 28 linear mixed models were computed to investigate the ER strategies (14 models for frequency and 14 models for efficacy) at time-point 3 in the separate groups. Interactions between age, gender, and group were also computed. Again, country and family identification code were used as random factors while we controlled for age and gender.

A set of exploratory analyses were computed wherein we attempted to investigate anxiety, concerns and ER strategies using generalised linear mixed modelling (GLMER). Outputs from the exploratory analyses are presented in Supplementary Materials B. Differences between the LMM and the GLMER models are noted in the results and discussion.

To correct for Type-I error due to the large sample size, we reduced the significance level from α = 0.05 to α = 0.01. Pre-processing of the data was conducted using MATLAB software. Analyses were conducted using R statistical software version 4.0.3^62^, with the packages *lme4* and *lmerTest* for multilevel regression^63,64^, *emmeans*^65^ and *multcomp*^66^ for pairwise comparisons, and *ggplot2*^67^ for visualization of main effects of the model.

## Results

### Anxiety of individuals with DS and WS

Descriptive analyses showed that the range of anxiety for individuals with DS and WS was minimum 1 and maximum 5. The median score for those with DS was 1 (*M* = 1.87, *SD* = 1.13) and for those with WS was 3 (*M* = 2.74, *SD* = 1.15).

**Figure 1:**
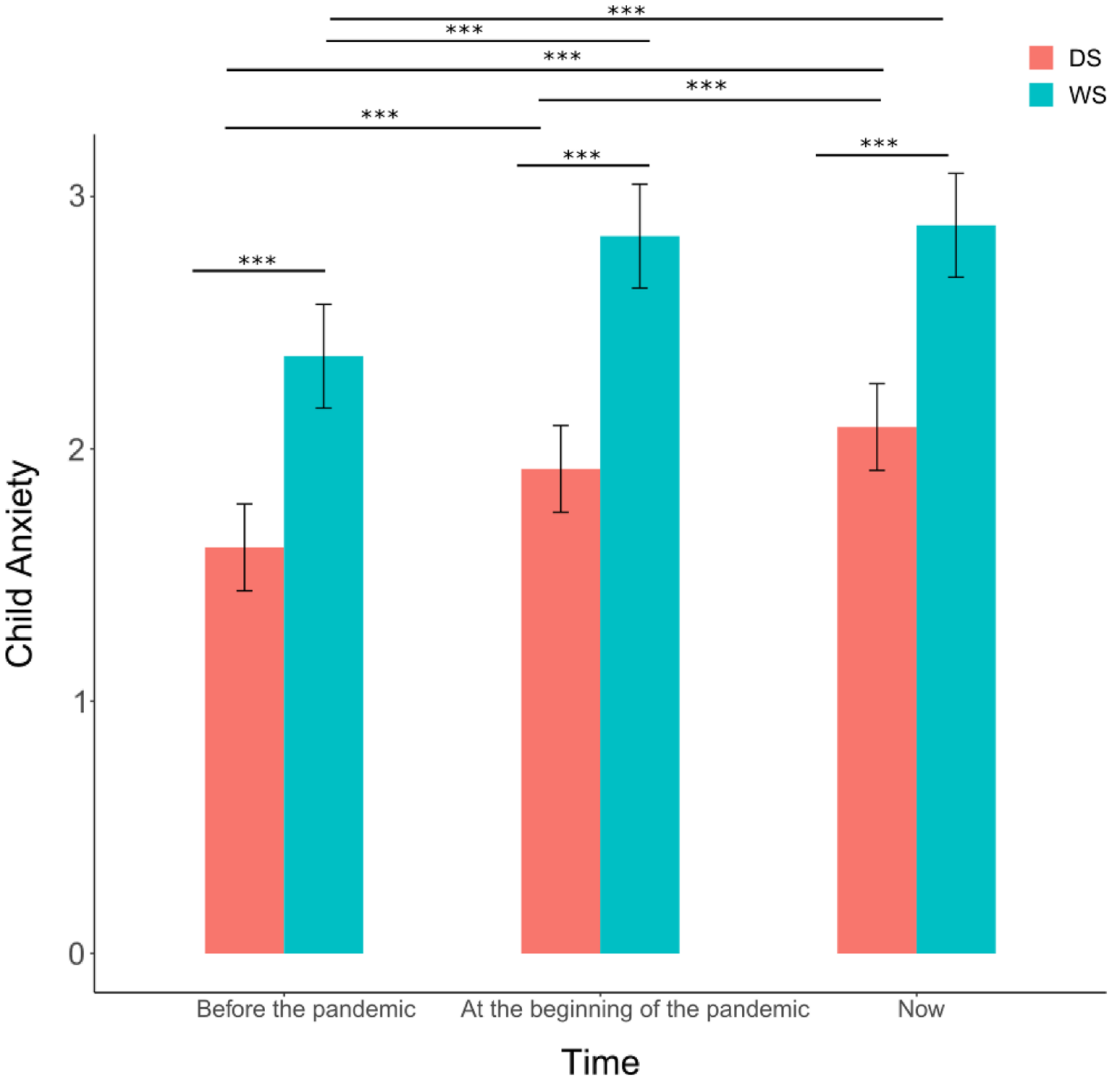
Mean anxiety of young people with Williams Syndrome (WS) and Down syndrome (DS) at three time points: before the pandemic, at the beginning of the pandemic and in the now moment (between April and August 2020). Error bars represent confidence intervals at 95%. Significance levels of post-hoc tests: *** = *p* < .001.

The analyses of anxiety levels revealed a main effect of group; *F (*1,654) = 88.07, *p* < 0.001, Time; *F (*2,1607) = 139.11,* p* < 0.2.2e−16, age; *F (*1,801) = 17.06, *p* < 0.3.992e−05, indicating increased anxiety with increasing age, no interaction between Time and group*; F (*2,1607) = 3.38, *p* = 0.034 and no effect for gender; *F (*1,794) = 0.04, *p* = 0.84. Post-hoc tests revealed significant differences between individuals with DS and WS at each of the three time-points (β = − 0.76, *t* = − 7.90, *p* < 0.001; *β* = − 0.92, *t* = − 9.62*, p* < 0.001; *β* = − 0.80, *t* = -8.32*, p* < 0.001), showing that those with WS experienced higher levels of anxiety than those with DS (see Fig. 1). Moreover, post-hoc tests revealed significant differences between the three time-points for the DS group, specifically between before the pandemic and at the beginning of the pandemic (*β* = − 0.31, *t* = − 8.50, *p* < 0.001), as well as between before the pandemic and the now moment (*β* = − 0.48, *t* = − 13.04, *p* < 0.001), and between at the beginning of the pandemic and the now moment (*β* = − 0.17,* t* = − 4.54, *p* < 0.001). Whilst the exploratory analysis of anxiety using GLMER showed no differences in the main effects, there was a key difference for one of post-hoc contrasts. The GLMER model revealed no significant difference for anxiety in individuals with DS between the beginning of the pandemic and the now moment (*β* = − 0.08,* Z* = − 2.00, *p* = *0.11*).

On the other hand, for the WS group post-hoc tests revealed significant differences only for two time-points, specifically for the before pandemic and at the beginning of the pandemic (*β* = − 0.47, *t* = − 8.67, *p* < 0.001), as well as between before the pandemic and the now moment (β = − 0.52, *t* = -9.44, *p* < 0.001) but no significant difference was observed between the beginning of the pandemic and the now moment (*β* = − 0.04, *t* = − 078, *p* = 0.71) (see Fig. 1). There were no differences in the significance of the results between the LMM and GLMER models.

### Main concerns in the two groups

On a descriptive level, when sorting the concerns for both groups according to their frequency (see supplementary material C), it can be observed that the highest concerns for individuals with WS were loss of social contact, not being able to approach others, loss of routine, and that others will get ill with COVID-19. For those with DS, the highest concerns were about the loss of social contact, loss of routine, not begin able to approach others and loss of institutional support.

**Figure 2:**
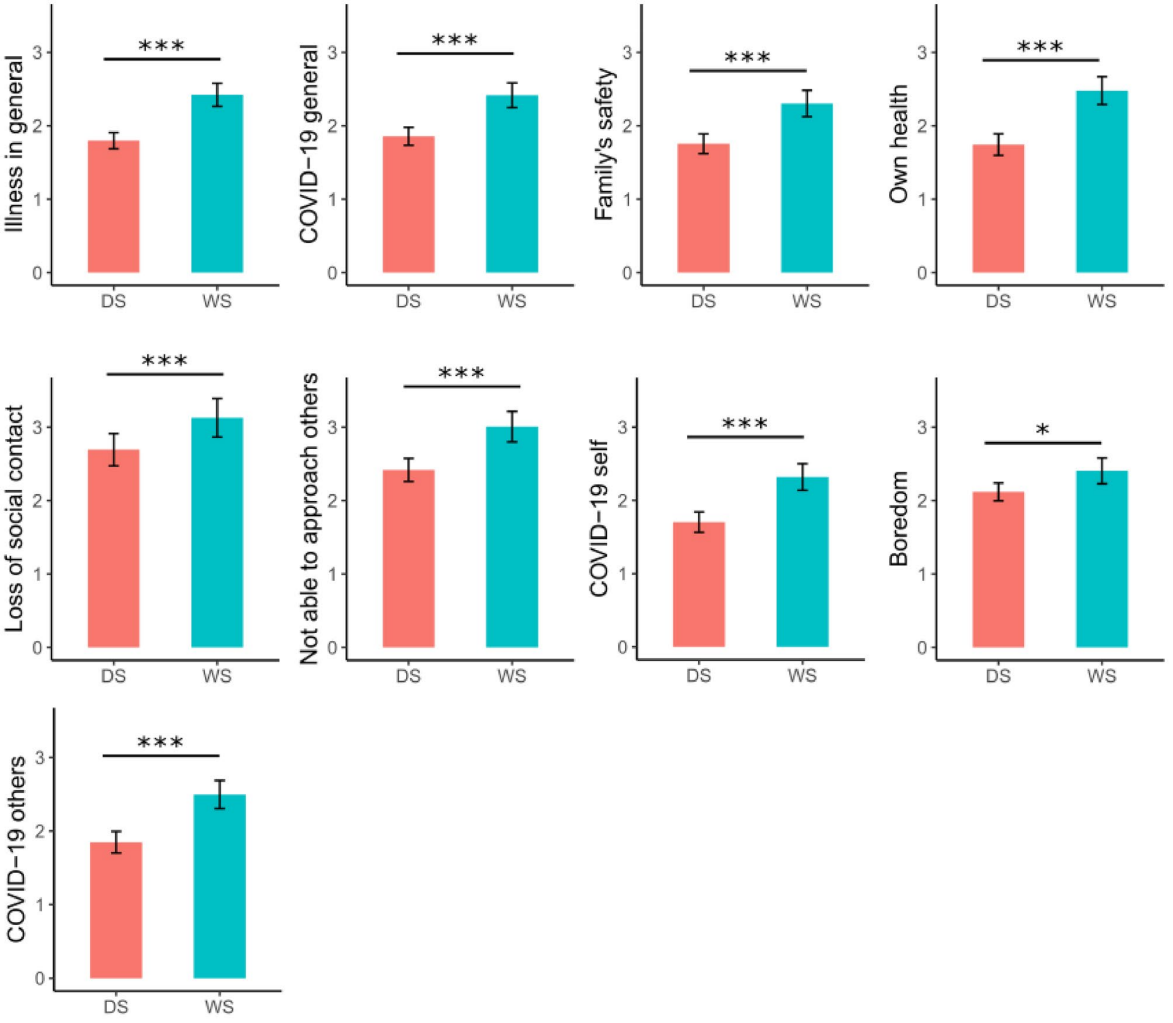
Group differences in level of different concerns between young people with Down syndrome (DS) and Williams syndrome (WS). Mean levels with confidence intervals are shown for each different concern. Significant effects of post hoc tests are indicated with black lines. Significance levels of post-hoc tests: * = *p* < .01, *** = *p* < .001.

The analyses of concerns revealed significant group effects in 9 out of 13 concerns (see Fig. 2), showing consistently higher concerns in individuals with WS compared to those with DS. No significant group differences were found for concerns related to loss of routine; loss of institutional support; family conflict and financial support. No gender effects were found. There was an age effect for 9 out of 13 concerns (see Table 1) indicating that the concerns increased with increasing age except for loss of routine; boredom; loss of institutional support and family conflict, where no age effect was found. However, no gender effects were detected. For the statistics see Table 1. A significant Gender*Group interaction was revealed for concerns relating to COVID-19 others: *F (*1,784) = 7.75, *p* = 0.01. Table 2 presents all the estimates. You can find the visualisation of the significant interaction in Supplementary Material D. There were no differences in the significance of the results between the LMM and GLMER models.

**Table 1:** Effects of group, age, and gender within the multilevel analyses on concerns.

Concerns	Group	Age	Age*group	Gender	Gender*group	Gender*Age	Gender*age*group
Illness in general	*F*(1,141) = 39.77, *p* = 3.47e−09	*F*(1,751) = 50.99, *p* = 2.2e−12	*F*(1,792) = 0.04, *p* = 0.84	*F*(1,790) = 1.18, *p* = 0.28	*F*(1,791) = 2.67, *p* = 0.10	*F*(1,792) = 0.82, *p* = 0.37	*F*(1,791) = 1.44, *p* = 0.23
COVID-19 general	*F*(1,137) = 28.74, *p* = 3.41e−16	*F*(1,750) = 67.53, *p* = 9.11e−16	*F*(1,792) = 0.87, *p* = 0.35	*F*(1,790) = 0.89, *p* = 0.35	*F*(1,792) = 4.37, *p* = 0.04	*F*(1,793) = 0.25, *p* = 0.62	*F*(1,792) = 0.06, *p* = 0.81
Family’s safety	*F*(1,224) = 26.6, *p* = 5.5e−07	*F*(1,756) = 47.57, *p* = 1.12e−11	*F*(1,791) = 0.23, *p* = 0.63	*F*(1,787) = 0.57, *p* = 0.45	*F*(1,788) = 3.21, *p* = 0.07	*F*(1,791) = 0.42, *p* = 0.51	*F*(1,789) = 0, *p* = 0.96
Own health	*F*(1,353) = 49.79, *p* = 9.12e−12	*F*(1,767) = 47.27, *p* = 1.28e−11	*F*(1,791) = 0.05, *p* = 0.82	*F*(1,786) = 0.82, *p* = 0.37	*F*(1,788) = 2.62, *p* = 0.11	*F*(1,791) = 0.23, *p* = 0.63	*F*(1,789) = 1.39, *p* = 0.24
Loss of social contact	*F*(1,580) = 12.63, *p* = 0.00	*F*(1,784) = 7.08, *p* = 0.01	*F*(1,786) = 4.75, *p* = 0.03	*F*(1,782) = 0.77, *p* = 0.38	*F*(1,783) = 0.03, *p* = 0.87	*F*(1,786) = 0.23, *p* = 0.63	*F*(1,783) = 0.11, *p* = 0.74
Not able to approach others	*F*(1,241) = 25.07, *p* = 1.07e−06	*F*(1,755) = 14, *p* = 0.00	*F*(1,790) = 5.42, *p* = 0.02	*F*(1,785) = 0.4, *p* = 0.53	*F*(1,787) = 0, *p* = 0.95	*F*(1,789) = 0.67, *p* = 0.41	*F*(1,788) = 0.02, *p* = 0.89
Loss of routine	*F*(1,448) = 4.22, *p* = 0.04	*F*(1,773) = 5.71, *p* = 0.02	*F*(1,788) = 0.04, *p* = 0.83	*F*(1,783) = 0.17, *p* = 0.68	*F*(1,785) = 0.11, *p* = 0.74	*F*(1,788) = 1, *p* = 0.32	*F*(1,786) = 0.6, *p* = 0.44
Boredom	*F*(1,112) = 6.41, *p* = 0.01	*F*(1,745) = 5.29, *p* = 0.02	*F*(1,787) = 0.01, *p* = 0.93	*F*(1,786) = 0.02, *p* = 0.89	*F*(1,787) = 0.09, *p* = 0.76	*F*(1,788) = 0.03, *p* = 0.86	*F*(1,788) = 0.1, *p* = 0.75
COVID-19 self	*F*(1,286) = 35.09, *p* = 9.01e−09	*F*(1,758) = 52.19, *p* = 1.23e−12	*F*(1,788) = 0.08, *p* = 0.77	*F*(1,783) = 0.09, *p* = 0.77	*F*(1,785) = 2.76, *p* = 0.1	*F*(1,787) = 0.28, *p* = 0.60	*F*(1,786) = 1.32, *p* = 0.25
COVID-19 others	*F*(1,275) = 35.84, *p* = 6.67e−09	*F*(1,757) = 49.98, *p* = 3.54e−12	*F*(1,787) = 3.44, *p* = 0.06	*F*(1,782) = 0.1, *p* = 0.75	*F*(1,784) = 7.75, *p* = 0.01	*F*(1,786) = 0.55, *p* = 0.46	*F*(1,786) = 0, *p* = 0.99
Loss of institutional support	*F*(1,502) = 0.09, *p* = 0.76	*F*(1,775) = 0.5, *p* = 0.48	*F*(1,786) = 0.01, *p* = 0.9	*F*(1,780) = 0.43, *p* = 0.51	*F*(1,782) = 0.04, *p* = 0.85	*F*(1,786) = 1.55, *p* = 0.21	*F*(1,783) = 0.01, *p* = 0.93
Family conflict	*F*(1,234) = 2.92, *p* = 0.09	*F*(1,755) = 0.17, *p* = 0.68	*F*(1,790) = 0.99, *p* = 0.32	*F*(1,786) = 0.28, *p* = 0.60	*F*(1,787) = 0.21, *p* = 0.65	*F*(1,790) = 1.18, *p* = 0.28	*F*(1,788) = 1.13, *p* = 0.32
Financial concerns	*F*(1,642) = 3.13, *p* = 0.08	*F*(1,785) = 6.92, *p* = 0.01	*F*(1,781) = 0.83, *p* = 0.36	*F*(1,777) = 4.71, *p* = 0.03	*F*(1,778) = 2.20, *p* = 0.14	*F*(1,782) = 0.13, *p* = 0.72	*F*(1,779) = 0.02, *p* = 0.88

**Table 2:** Estimates of group, age, and gender within the multilevel analyses on concerns.

Concerns	Group	Age	Age*Group	Gender	Gender*Group	Gender*Age	Gender*Age*Group
Illness in general	*β* = 0.79, t = 5.73, *p* = 2.43e−08	*β* = 0.39, t = 4.59, *p* = 5.24e−06	*β* = − 0.11, t = − 0.79, *p* = 0.43	*β* = − 0.01, t = − 0.06, *p* = 0.95	*β* = − 0.31, t = − 1.7, *p* = 0.09	*β* = − 0.16, t = − 1.45, *p* = 0.15	*β* = 0.21, t = 1.2, *p* = 0.23
COVID-19 general	*β* = 0.78, t = 5.44, *p* = 1.40e−07	*β* = 0.43, t = 4.83, *p* = 1.62e−06	*β* = − 0.11, t = − 0.79, *p* = 0.43	*β* = 0.04, t = 0.37, *p* = 0.71	*β* = − 0.4, t = − 2.1, *p* = 0.04	*β* = − 0.06, t = − 0.54, *p* = 0.59	*β* = 0.05, t = 0.24, *p* = 0.81
Family’s safe ty	*β* = 0.74, t = 5.03, *p* = 7.43e−07	*β* = 0.36, t = 4.02, *p* = 6.28e−05	*β* = − 0.05, t = − 0.34, *p* = 0.73	*β* = 0.04, t = 0.37, *p* = 0.71	*β* = − 0.34, t = − 1.79, *p* = 0.07	*β* = − 0.06, t = − 0.55, *p* = 0.59	*β* = 0.01, t = 0.04, *p* = 0.96
Own Health	*β* = 0.9, t = 6.26, *p* = 8.42e−10	*β* = 0.38, t = 4.37, *p* = 1.42e−05	*β* = − 0.15, t = − 1.06, *p* = 0.29	*β* = 0.01, t = 0.09, *p* = 0.93	*β* = − 0.31, t = − 1.68, *p* = 0.09	*β* = − 0.12, t = − 1.1, *p* = 0.27	*β* = 0.21, t = 1.18, *p* = 0.24
Loss of social con tac t	*β* = 0.43, t = 2.57, *p* = 0.01	*β* = 0.26, t = 2.57, *p* = 0.01	*β* = − 0.26, t = − 1.64, *p* = 0.1	*β* = − 0.1, t = − 0.84, *p* = 0.40	*β* = 0.03, t = 0.15, *p* = 0.88	*β* = − 0.07, t = − 0.58, *p* = 0.56	*β* = 0.07, t = 0.33, *p* = 0.74
Not able to approach others	*β* = 0.61, t = 3.69, *p* = 0–00	*β* = 0.32, t = 3.16, *p* = 0.00	*β* = − 0.22, t = − 1.39, *p* = 0.17	*β* = − 0.07, t = − 0.56, *p* = 0.58	*β* = 0.01, t = 0.06, *p* = 0.95	*β* = − 0.07, t = − 0.57, *p* = 0.57	*β* = − 0.03, t = − 0.13, *p* = 0.89
Loss of routine	*β* = 0.22, t = 1.27, *p* = 0.20	*β* = 0.15, t = 1.49, *p* = 0.14	*β* = 0.08, t = 0.46, *p* = 0.64	*β* = 0.02, t = 0.18, *p* = 0.86	*β* = 0.08, t = 0.37, *p* = 0.71	*β* = − 0.04, t = − 0.31, *p* = 0.76	*β* = − 0.17, t = − 0.78, *p* = 0.44
Boredom	*β* = 0.25, t = 1.61, *p* = 0.11	*β* = 0.14, t = 1.43, *p* = 0.15	*β* = − 0.05, t = − 0.31, *p* = 0.76	*β* = − 0.04, t = − 0.31, *p* = 0.76	*β* = 0.06, t = 0.29, *p* = 0.77	*β* = − 0.04, t = − 0.34, *p* = 0.73	*β* = 0.07, t = 0.32, *p* = 0.75
COVID-19 self	*β* = 0.79, t = 5.44, *p* = 9.15e−08	*β* = 0.38, t = 4.34, *p* = 1.59e−05	*β* = − 0.1, t = − 0.7, *p* = 0.48	*β* = 0.11, t = 1.1, *p* = 0.27	*β* = − 0.32, t = − 1.72, *p* = 0.09	*β* = − 0.12, t = − 1.12, *p* = 0.26	*β* = 0.21, t = 1.15, *p* = 0.25
COVID-19 others	*β* = 0.95, t = 6.41, *p* = 3.42e−10	*β* = 0.42, t = 4.66, *p* = 3.69e−06	*β* = − 0.17, t = − 1.19, *p* = 0.24	*β* = 0.19, t = 1.8, *p* = 0.07	*β* = − 0.53, t = − 2.78, *p* = 0.01	*β* = − 0.07, t = − 0.6, *p* = 0.55	*β* = 0, t = 0.02, *p* = 0.99
Loss of institutional support	*β* = 0.06, t = 0.34, *p* = 0.73	*β* = 0.12, t = 1.1, *p* = 0.27	*β* = 0, t = 0.01, *p* = 0.99	*β* = 0.08, t = 0.61, *p* = 0.54	*β* = − 0.04, t = − 0.19, *p* = 0.85	*β* = − 0.14, t = − 1.04, *p* = 0.30	*β* = 0.02, t = 0.09, *p* = 0.93
Family conflict	*β* = 0.22, t = 1.62, *p* = 0.11	*β* = 0.06, t = 0.72, *p* = 0.47	*β* = 0.02, t = 0.18, *p* = 0.85	*β* = 0.07, t = 0.73, *p* = 0.46	*β* = − 0.07, t = − 0.41, *p* = 0.68	*β* = − 0.02, t = − 0.21, *p* = 0.83	*β* = − 0.19, t = − 1.07, *p* = 0.29
Financial concerns	*β* = 0.22, t = 2.27, *p* = 0.02	*β* = 0.07, t = 1.12, *p* = 0.26	*β* = 0.06, t = 0.7, *p* = 0.49	*β* = 0.18, t = 2.63, *p* = 0.01	*β* = − 0.18, t = − 1.47, *p* = 0.14	*β* = − 0.01, t = − 0.2, *p* = 0.84	*β* = − 0.02, t = − 0.15, *p* = 0.88

### Differences in ER frequency and efficacy in the two groups

#### Frequency of emotion regulation strategies

**Figure 3:**
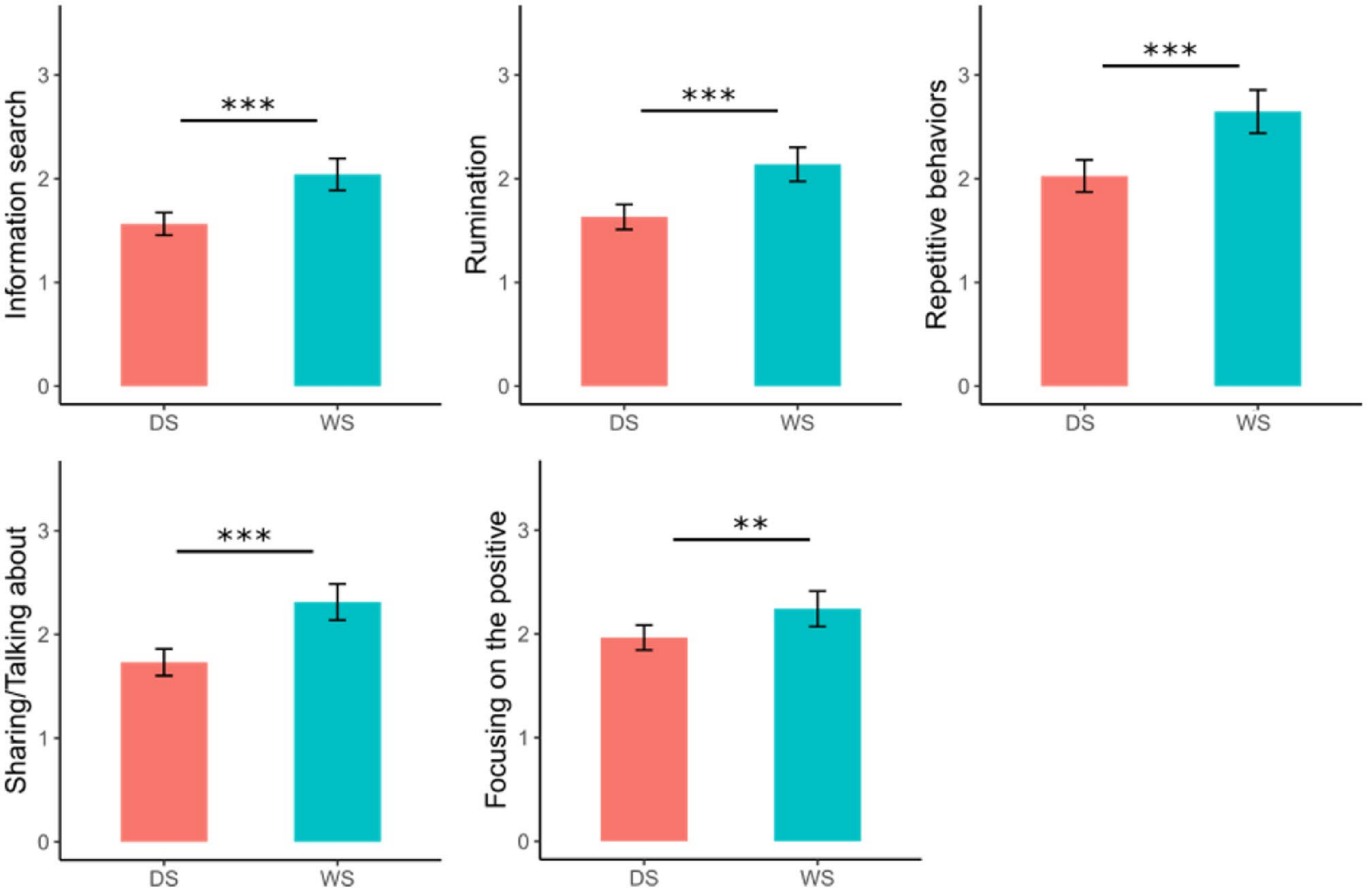
Significant group differences in the frequency of different ER strategies between young people with Down syndrome (DS) and Williams syndrome (WS). Mean levels with confidence intervals are shown for each ER strategies. Significant effects of post hoc tests are indicated (black lines). Significance levels of post-hoc tests: ** = *p* < .01, *** = *p* < .001.

We found significant effects of group indicating a more frequent use of ER strategies in those with WS in the following strategies: searching for more information, rumination, repetitive behaviours, sharing/talking about COVID-19 and focusing on the positive. Table 3 presents group, age, gender, and all computed interactions outputs. No gender differences in the frequency of ER strategies between individuals with DS or WS. However, for all strategies usage there was an increase with age (see Table 3 and Fig. 3). A significant Age*Group interaction was revealed for ER strategy for humour: *F (1,796)* = *7.43. p* = 0.01 (see Supplementary Material E for the visualisation of the interaction). Table 4 presents the estimates for group, age, gender, and all computed interactions. There were no differences in the significance of the results between the LMM and GLMER models.

**Table 3:** Effects of group, age, and gender within the multilevel analyses on emotion regulation (ER) frequency.

ER Strategy (Frequency)	Group	Age	Age*group	Gender	Gender*group	Gender*age	Gender*Age*group
Isolation/Withdrawal	*F* (1,128) = 1.04, *p* = 0.31	*F* (1,756) = 26.5, *p* = 3.35e−07	*F* (1,796) = 0.74, *p* = 0.39	*F* (1,794) = 3.39, *p* = 0.07	*F* (1,795) = 0.61, *p* = 0.44	*F* (1,796) = 0.26, *p* = 0.61	*F* (1,795) = 2.53, *p* = 0.11
Information Avoidance	*F* (1,72) = 0.02, *p* = 0.88	*F* (1,751) = 15.88, *p* = 7.42e−05	*F* (1,793) = 0.69, *p* = 0.41	*F* (1,794) = 1.72, *p* = 0.19	*F* (1,795) = 0.22, *p* = 0.64	*F* (1,796) = 5.09, *p* = 0.02	*F* (1,796) = 0.02, *p* = 0.87
Information Search	*F* (1,183) = 24.82, *p* = 1.45e−06	*F* (1,759) = 8.68, *p* = 0.01	*F* (1,796) = 2.03, *p* = 0.15	*F* (1,793) = 0.36, *p* = 0.55	*F* (1,794) = 1.38, *p* = 0.24	*F* (1,796) = 0.28, *p* = 0.59	*F* (1,795) = 0.37, *p* = 0.55
Rumination	*F* (1,207) = 26.93, *p* = 5.03e−07	*F* (1,761) = 24.54, *p* = 8.98e−07	*F* (1,796) = 0.58, *p* = 0.45	*F* (1,792) = 0.56, *p* = 0.45	*F* (1,794) = 0.42, *p* = 0.52	*F* (1,796) = 0.04, *p* = 0.83	*F* (1,795) = 0.79, *p* = 0.37
Expressive suppression	*F* (1,232) = 0.2, *p* = 0.66	*F* (1,762) = 9.08, *p* = 0.01	*F* (1,796) = 1.47, *p* = 0.23	*F* (1,792) = 0.02, *p* = 0.88	*F* (1,793) = 0.46, *p* = 0.50	*F* (1,796) = 0.18, *p* = 0.67	*F* (1,794) = 2.55, *p* = 0.11
Aggressive behaviours	*F* (1,149) = 1.24, *p* = 0.27	*F* (1,758) = 14.39, *p* = 0.00	*F* (1,796) = 0.19, *p* = 0.66	*F* (1,793) = 3.97, *p* = 0.05	*F* (1,794) = 0.19, *p* = 0.67	*F* (1,796) = 0.91, *p* = 0.34	*F* (1,795) = 3.36, *p* = 0.07
Repetitive Behaviours	*F* (1,195) = 25.44, *p* = 1.04e−06	*F* (1,760) = 1, *p* = 0.32	*F* (1,796) = 3.89, *p* = 0.05	*F* (1,793) = 4.57, *p* = 0.03	*F* (1,794) = 1.59, *p* = 0.21	*F* (1,796) = 2.28, *p* = 0.13	*F* (1,795) = 0.58, *p* = 0.45
Sharing/Talking about COVID-19	*F* (1,217) = 31.33, *p* = 6.54e−08	*F* (1,761) = 2.2, *p* = 0.14	*F* (1,796) = 5.69, *p* = 0.02	*F* (1,792) = 0.81, *p* = 0.37	*F* (1,794) = 0.75, *p* = 0.39	*F* (1,796) = 0.24, *p* = 0.62	*F* (1,794) = 0.02, *p* = 0.89
Distraction	*F* (1,312) = 5.13, *p* = 0.02	*F* (1,768) = 8.95, *p* = 0.00	*F* (1,796) = 0.15, *p* = 0.70	*F* (1,791) = 2.22, *p* = 0.14	*F* (1,792) = 1.54, *p* = 0.22	*F* (1,795) = 3.06, *p* = 0.08	*F* (1,793) = 0.2, *p* = 0.66
Cognitive reappraisal	*F* (1,368) = 4.14, *p* = 0.04	*F* (1,773) = 13.15, *p* = 0.00	*F* (1,795) = 1.02, *p* = 0.31	*F* (1,790) = 1.35, *p* = 0.25	*F* (1,792) = 0.32, *p* = 0.57	*F* (1,795) = 5.42, *p* = 0.02	*F* (1,793) = 0.21, *p* = 0.65
Focusing on the positive	*F* (1,142) = 6.18, *p* = 0.01	*F* (1,757) = 4.9, *p* = 0.03	*F* (1,796) = 1.81, *p* = 0.18	*F* (1,793) = 0.78, *p* = 0.38	*F* (1,795) = 0.03, *p* = 0.86	*F* (1,796) = 5.85, *p* = 0.02	*F* (1,795) = 0.03, *p* = 0.86
Humour	*F* (1,297) = 4.78, *p* = 0.03	*F* (1,767) = 1.21, *p* = 0.27	*F* (1,796) = 7.43, *p* = 0.01	*F* (1,791) = 2.3, *p* = 0.13	*F* (1,793) = 1.29, *p* = 0.26	*F* (1,795) = 2.3, *p* = 0.13	*F* (1,793) = 0, *p* = 0.98
Parent shielding	*F* (1,739) = 4.28, *p* = 0.04	*F* (1,796) = 0.08, *p* = 0.78	*F* (1,787) = 0.11, *p* = 0.74	*F* (1,783) = 0, *p* = 0.99	*F* (1,784) = 2.82, *p* = 0.09	*F* (1,788) = 0.08, *p* = 0.78	*F* (1,785) = 1.29, *p* = 0.26
Parent routine	*F* (1,686) = 0.07, *p* = 0.79	*F* (1,796) = 0.79, *p* = 0.37	*F* (1,789) = 2.38, *p* = 0.12	*F* (1,785) = 4.3, *p* = 0.04	*F* (1,786) = 0, *p* = 0.98	*F* (1,790) = 0.18, *p* = 0.67	*F* (1,787) = 0.21, *p* = 0.65

**Table 4:** Estimates of group, age, gender and interactions within the multilevel analyses on emotion regulation (ER) frequency.

ER strategy (Frequency)	Group	Age	Age*Group	Gender	Gender*Group	Gender*Age	Gender*Age*Group
Isolation/Withdrawal	*β* = − 0.19,t = − 1.25, *p* = 0.21	*β* = 0.31,t = 3.31, *p* = 0.00	*β* = − 0.27,t = − 1.78, *p* = 0.08	*β* = − 0.23,t = − 2.04, *p* = 0.04	*β* = 0.14,t = 0.7, *p* = 0.48	*β* = − 0.07,t = − 0.57, *p* = 0.57	*β* = 0.31,t = 1.59, *p* = 0.11
Information avoidance	*β* = − 0.03,t = − 0.19, *p* = 0.85	*β* = 0.32,t = 3.72, *p* = 0.00	*β* = − 0.09,t = − 0.65, *p* = 0.51	*β* = − 0.14,t = − 1.39, *p* = 0.17	*β* = 0.09,t = 0.46, *p* = 0.64	*β* = − 0.21,t = − 1.89, *p* = 0.06	*β* = 0.03,t = 0.16, *p* = 0.87
Information search	*β* = 0.38,t = 2.83, *p* = 0.01	*β* = 0.21,t = 2.59, *p* = 0.01	*β* = − 0.18,t = − 1.38, *p* = 0.17	*β* = − 0.02,t = − 0.2, *p* = 0.84	*β* = 0.2,t = 1.14, *p* = 0.25	*β* = − 0.08,t = − 0.79, *p* = 0.43	*β* = 0.1,t = 0.61, *p* = 0.54
Rumination	*β* = 0.58,t = 4.25, *p* = 2.49e−05	*β* = 0.27,t = 3.28, *p* = 0.00	*β* = − 0.16,t = − 1.17, *p* = 0.24	*β* = 0.09,t = 0.93, *p* = 0.35	*β* = − 0.12,t = − 0.69, *p* = 0.49	*β* = − 0.07,t = − 0.71, *p* = 0.48	*β* = 0.15,t = 0.89, *p* = 0.37
Expressive suppression	*β* = 0.09,t = 0.73, *p* = 0.47	*β* = 0.16,t = 2.07, *p* = 0.04	*β* = − 0.06,t = − 0.45, *p* = 0.65	*β* = 0.04,t = 0.41, *p* = 0.68	*β* = − 0.13,t = − 0.76, *p* = 0.45	*β* = − 0.13,t = − 1.32, *p* = 0.19	*β* = 0.26,t = 1.6, *p* = 0.11
Aggressive behaviours	*β* = 0.07,t = 0.52, *p* = 0.60	*β* = − 0.17,t = − 2.01, *p* = 0.04	*β* = 0.15,t = 1.13, *p* = 0.26	*β* = 0.15,t = 1.49, *p* = 0.14	*β* = 0.09,t = 0.52, *p* = 0.60	*β* = 0.04,t = 0.36, *p* = 0.72	*β* = − 0.31,t = − 1.83, *p* = 0.07
Repetitive Behaviours	*β* = 0.48,t = 2.84, *p* = 0.00	*β* = 0.08,t = 0.78, *p* = 0.43	*β* = − 0.11,t = − 0.67, *p* = 0.50	*β* = 0.13,t = 1.08, *p* = 0.28	*β* = 0.29,t = 1.3, *p* = 0.19	*β* = − 0.1,t = − 0.74, *p* = 0.46	*β* = − 0.17,t = − 0.76, *p* = 0.44
Sharing/Talking about COVID-19	*β* = 0.51,t = 3.55, *p* = 0.00	*β* = 0.12,t = 1.37, *p* = 0.17	*β* = − 0.23,t = − 1.63, *p* = 0.10	*β* = − 0.13,t = − 1.23, *p* = 0.22	*β* = 0.16,t = 0.86, *p* = 0.39	*β* = 0.03,t = 0.31, *p* = 0.76	*β* = 0.02,t = 0.13, *p* = 0.89
Distraction	*β* = 0.12,t = 0.78, *p* = 0.44	*β* = 0.24,t = 2.47, *p* = 0.01	*β* = 0.01,t = 0.1, *p* = 0.92	*β* = − 0.22,t = − 1.93, *p* = 0.05	*β* = 0.26,t = 1.26, *p* = 0.21	*β* = − 0.14,t = − 1.12, *p* = 0.26	*β* = − 0.09,t = − 0.44, *p* = 0.66
Cognitive reappraisal	*β* = 0.13,t = 1.13, *p* = 0.26	*β* = 0.26,t = 3.8, *p* = 0.00	*β* = − 0.11,t = − 1, *p* = 0.32	*β* = − 0.11,t = − 1.33, *p* = 0.18	*β* = 0.08,t = 0.54, *p* = 0.59	*β* = − 0.19,t = − 2.13, *p* = 0.03	*β* = 0.07,t = 0.46, *p* = 0.64
Focusing on the positive	*β* = 0.26,t = 1.75, *p* = 0.08	*β* = 0.29,t = 3.14, *p* = 0.00	*β* = − 0.15,t = − 1, *p* = 0.32	*β* = − 0.1,t = − 0.87, *p* = 0.39	*β* = 0.03,t = 0.16, *p* = 0.87	*β* = − 0.24,t = − 2.03, *p* = 0.04	*β* = 0.03,t = 0.18, *p* = 0.86
Humour	*β* = 0.13,t = 0.92, *p* = 0.36	*β* = 0.21,t = 2.48, *p* = 0.01	*β* = − 0.24,t = − 1.76, *p* = 0.08	*β* = 0.06,t = 0.6, *p* = 0.55	*β* = 0.21,t = 1.13, *p* = 0.26	*β* = − 0.13,t = − 1.22, *p* = 0.22	*β* = 0,t = 0.02, *p* = 0.98
Parent shielding	*β* = 0.47,t = 2.67, *p* = 0.01	*β* = − 0.07,t = − 0.7, *p* = 0.48	*β* = 0.18,t = 1.09, *p* = 0.28	*β* = 0.12,t = 1.01, *p* = 0.31	*β* = − 0.35,t = − 1.62, *p* = 0.11	*β* = 0.12,t = 0.92, *p* = 0.36	*β* = − 0.24,t = − 1.14, *p* = 0.26
Parent routine	*β* = − 0.02,t = − 0.13, *p* = 0.9	*β* = 0.02,t = 0.16, *p* = 0.88	*β* = − 0.11,t = − 0.64, *p* = 0.52	*β* = 0.21,t = 1.72, *p* = 0.09	*β* = 0.01,t = 0.05, *p* = 0.96	*β* = − 0.01,t = − 0.06, *p* = 0.96	*β* = − 0.1,t = − 0.45, *p* = 0.65

### Efficacy of emotion regulation strategies

No group or age effects in the efficacy of ER strategies were observed. However, we found gender effects for isolation/withdrawal, information avoidance, and sharing/talking about COVID-19 and distraction, in the sense that these strategies were less efficiently used by male participants. Tables 5 and 6 present the group, age, gender and all computed interactions effects and estimates. No significant interactions were revealed. There were no differences in the significance of the results between the LMM and GLMER models.

**Table 5:** Effects of group, age, and gender within the multilevel analyses on emotion regulation (ER) efficacy.

ER strategy (Efficacy)	Group	Age	Age*Group	Gender	Gender*Group	Gender*Age	Gender*Age*Group
Isolation/withdrawal	*F* (1,296) = 5.54, *p* = 0.02	*F* (1,755) = 4.78, *p* = 0.03	*F* (1,782) = 2.76, *p* = 0.10	*F* (1,778) = 7, *p* = 0.01	*F* (1,779) = 0.24, *p* = 0.62	*F* (1,781) = 1.98, *p* = 0.16	*F* (1,780) = 1.36, *p* = 0.24
Information avoidance	*F* (1,298) = 0.06, *p* = 0.8	*F* (1,759) = 2.01, *p* = 0.16	*F* (1,788) = 1.94, *p* = 0.16	*F* (1,783) = 6.78, *p* = 0.01	*F* (1,785) = 0, *p* = 0.99	*F* (1,787) = 3.65, *p* = 0.06	*F* (1,785) = 0.03, *p* = 0.86
Information search	*F* (1,152) = 1.13, *p* = 0.29	*F* (1,750) = 0, *p* = 0.98	*F* (1,787) = 2.17, *p* = 0.14	*F* (1,784) = 2.88, *p* = 0.09	*F* (1,785) = 1.93, *p* = 0.17	*F* (1,787) = 0.21, *p* = 0.65	*F* (1,786) = 0.13, *p* = 0.72
Rumination	*F* (1,70) = 0, *p* = 0.95	*F* (1,738) = 1.45, *p* = 0.23	*F* (1,777) = 1.31, *p* = 0.25	*F* (1,778) = 1.2, *p* = 0.27	*F* (1,780) = 0.1, *p* = 0.75	*F* (1,780) = 0.26, *p* = 0.61	*F* (1,780) = 0.47, *p* = 0.49
Expressive suppression	*F* (1,69) = 0.45, *p* = 0.51	*F* (1,740) = 0.02, *p* = 0.88	*F* (1,780) = 0.57, *p* = 0.45	*F* (1,781) = 0.53, *p* = 0.47	*F* (1,782) = 0, *p* = 0.94	*F* (1,783) = 0.11, *p* = 0.74	*F* (1,783) = 1.25, *p* = 0.26
Aggressive behaviours	*F* (1,68) = 0.04, *p* = 0.84	*F* (1,735) = 0.01, *p* = 0.93	*F* (1,775) = 0.07, *p* = 0.79	*F* (1,777) = 0.76, *p* = 0.38	*F* (1,778) = 0.07, *p* = 0.79	*F* (1,779) = 1.15, *p* = 0.28	*F* (1,779) = 1.67, *p* = 0.20
Repetitive behaviours	*F* (1,137) = 4.49, *p* = 0.04	*F* (1,752) = 0.3, *p* = 0.58	*F* (1,779) = 0.91, *p* = 0.34	*F* (1,777) = 1.41, *p* = 0.24	*F* (1,778) = 0.07, *p* = 0.79	*F* (1,780) = 0.32, *p* = 0.57	*F* (1,779) = 0.58, *p* = 0.45
Sharing/talking about COVID-19	*F* (1,320) = 1.09, *p* = 0.3	*F* (1,761) = 0.16, *p* = 0.69	*F* (1,787) = 0.56, *p* = 0.46	*F* (1,782) = 6.74, *p* = 0.01	*F* (1,783) = 0, *p* = 0.95	*F* (1,786) = 0.17, *p* = 0.68	*F* (1,784) = 0.33, *p* = 0.57
Distraction	*F* (1,325) = 0.21, *p* = 0.64	*F* (1,762) = 0.74, *p* = 0.39	*F* (1,785) = 3.32, *p* = 0.07	*F* (1,781) = 8.33, *p* = 0.00	*F* (1,782) = 1.07, *p* = 0.30	*F* (1,785) = 1.16, *p* = 0.28	*F* (1,783) = 0.12, *p* = 0.73
Cognitive reappraisal	*F* (1,76) = 0.39, *p* = 0.53	*F* (1,742) = 0.36, *p* = 0.55	*F* (1,779) = 3.39, *p* = 0.07	*F* (1,779) = 1.71, *p* = 0.19	*F* (1,780) = 0.11, *p* = 0.74	*F* (1,781) = 1.03, *p* = 0.31	*F* (1,781) = 0.15, *p* = 0.70
Focusing on the positive	*F* (1,266) = 0, *p* = 0.94	*F* (1,755) = 0.2, *p* = 0.65	*F* (1,785) = 3.18, *p* = 0.08	*F* (1,780) = 2.56, *p* = 0.11	*F* (1,782) = 0, *p* = 0.94	*F* (1,784) = 2.56, *p* = 0.11	*F* (1,783) = 0.06, *p* = 0.81
Humour	*F* (1,203) = 0.01, *p* = 0.94	*F* (1,749) = 1.11, *p* = 0.29	*F* (1,785) = 3.82, *p* = 0.05	*F* (1,782) = 0.01, *p* = 0.93	*F* (1,783) = 1.45, *p* = 0.23	*F* (1,785) = 0.31, *p* = 0.58	*F* (1,783) = 0.18, *p* = 0.67
Parent shielding	*F* (1,621) = 1.05, *p* = 0.31	*F* (1,780) = 0.56, *p* = 0.46	*F* (1,777) = 1.64, *p* = 0.2	*F* (1,772) = 2.76, *p* = 0.10	*F* (1,773) = 1.58, *p* = 0.21	*F* (1,778) = 0.49, *p* = 0.49	*F* (1,774) = 1.71, *p* = 0.19
Parent routine	*F* (1,609) = 0.6, *p* = 0.44	*F* (1,784) = 1.03, *p* = 0.31	*F* (1,781) = 1.87, *p* = 0.17	*F* (1,775) = 0.41, *p* = 0.52	*F* (1,776) = 0.19, *p* = 0.66	*F* (1,780) = 2.66, *p* = 0.10	*F* (1,777) = 2.13, *p* = 0.15

*p < .05. **p < .01. ***p < .001.

**Table 6:** Estimates of group, age, gender and interactions within the multilevel analyses on emotion regulation (ER) efficacy.

ER strategy (efficacy)	Group	Age	Age*group	Gender	Gender*group	Gender*age	Gender*age*group
Isolation/Withdrawal	*β* = − 0.34,t = − 1.96, *p* = 0.05	*β* = 0.15,t = 1.41, *p* = 0.16	*β* = − 0.33,t = − 1.96, *p* = 0.05	*β* = − 0.31,t = − 2.5, *p* = 0.01	*β* = 0.1,t = 0.43, *p* = 0.67	*β* = 0.05,t = 0.4, *p* = 0.69	*β* = 0.25,t = 1.17, *p* = 0.24
Information avoidance	*β* = 0.04,t = 0.24, *p* = 0.81	*β* = 0.23,t = 2.29, *p* = 0.02	*β* = − 0.12,t = − 0.76, *p* = 0.45	*β* = − 0.26,t = − 2.18, *p* = 0.03	*β* = 0.01,t = 0.03, *p* = 0.98	*β* = − 0.18,t = − 1.41, *p* = 0.16	*β* = − 0.04,t = − 0.17, *p* = 0.86
Information search	*β* = − 0.02,t = − 0.15, *p* = 0.88	*β* = 0.07,t = 0.66, *p* = 0.51	*β* = − 0.11,t = − 0.67, *p* = 0.5	*β* = − 0.26,t = − 2.17, *p* = 0.03	*β* = 0.3,t = 1.41, *p* = 0.16	*β* = − 0.02,t = − 0.14, *p* = 0.89	*β* = − 0.07,t = − 0.36, *p* = 0.72
Rumination	*β* = 0.04,t = 0.25, *p* = 0.8	*β* = 0.16,t = 1.66, *p* = 0.1	*β* = − 0.19,t = − 1.26, *p* = 0.21	*β* = − 0.09,t = − 0.77, *p* = 0.44	*β* = − 0.07,t = − 0.35, *p* = 0.73	*β* = − 0.1,t = − 0.82, *p* = 0.41	*β* = 0.14,t = 0.68, *p* = 0.49
Expressive suppression	*β* = − 0.08,t = − 0.5, *p* = 0.62	*β* = 0.11,t = 1.09, *p* = 0.27	*β* = − 0.21,t = − 1.34, *p* = 0.18	*β* = − 0.08,t = − 0.71, *p* = 0.48	*β* = 0,t = 0.02, *p* = 0.99	*β* = − 0.12,t = − 0.94, *p* = 0.35	*β* = 0.23,t = 1.12, *p* = 0.26
Aggressive behaviours	*β* = − 0.06,t = − 0.37, *p* = 0.71	*β* = 0,t = − 0.02, *p* = 0.98	*β* = 0.19,t = 1.17, *p* = 0.24	*β* = − 0.1,t = − 0.82, *p* = 0.41	*β* = 0.07,t = 0.33, *p* = 0.74	*β* = − 0.01,t = − 0.06, *p* = 0.95	*β* = − 0.28,t = − 1.3, *p* = 0.2
Repetitive behaviours	*β* = 0.24,t = 1.39, *p* = 0.17	*β* = 0.01,t = 0.06, *p* = 0.95	*β* = 0,t = − 0.03, *p* = 0.98	*β* = 0.11,t = 0.86, *p* = 0.39	*β* = 0.07,t = 0.31, *p* = 0.76	*β* = 0,t = 0.02, *p* = 0.98	*β* = − 0.17,t = − 0.76, *p* = 0.44
Sharing/Talking about COVID-19	*β* = 0.14,t = 0.84, *p* = 0.4	*β* = − 0.01,t = − 0.05, *p* = 0.96	*β* = − 0.01,t = − 0.04, *p* = 0.97	*β* = − 0.24,t = − 2.08, *p* = 0.04	*β* = − 0.01,t = − 0.03, *p* = 0.98	*β* = 0.08,t = 0.68, *p* = 0.5	*β* = − 0.12,t = − 0.57, *p* = 0.57
Distraction	*β* = − 0.05,t = − 0.29, *p* = 0.77	*β* = 0.17,t = 1.59, *p* = 0.11	*β* = − 0.15,t = − 0.9, *p* = 0.37	*β* = − 0.37,t = − 2.96, *p* = 0.00	*β* = 0.23,t = 1.05, *p* = 0.29	*β* = − 0.08,t = − 0.64, *p* = 0.52	*β* = − 0.08,t = − 0.35, *p* = 0.73
Cognitive reappraisal	*β* = 0.05,t = 0.31, *p* = 0.75	*β* = 0.08,t = 0.82, *p* = 0.41	*β* = − 0.14,t = − 0.88, *p* = 0.38	*β* = − 0.15,t = − 1.26, *p* = 0.21	*β* = 0.07,t = 0.35, *p* = 0.73	*β* = − 0.07,t = − 0.57, *p* = 0.57	*β* = − 0.08,t = − 0.38, *p* = 0.7
Focusing on the positive	*β* = 0,t = 0, *p* = 1	*β* = 0.14,t = 1.29, *p* = 0.2	*β* = − 0.16,t = − 0.96, *p* = 0.34	*β* = − 0.17,t = − 1.37, *p* = 0.17	*β* = 0.02,t = 0.08, *p* = 0.93	*β* = − 0.15,t = − 1.13, *p* = 0.26	*β* = − 0.05,t = − 0.24, *p* = 0.81
Humour	*β* = − 0.12,t = − 0.7, *p* = 0.48	*β* = 0.03,t = 0.33, *p* = 0.74	*β* = − 0.15,t = − 0.92, *p* = 0.36	*β* = − 0.09,t = − 0.73, *p* = 0.46	*β* = 0.27,t = 1.23, *p* = 0.22	*β* = − 0.02,t = − 0.18, *p* = 0.86	*β* = − 0.09,t = − 0.43, *p* = 0.67
Parent shielding	*β* = 0.3,t = 1.7, *p* = 0.09	*β* = − 0.1,t = − 0.95, *p* = 0.34	*β* = 0.03,t = 0.19, *p* = 0.85	*β* = − 0.07,t = − 0.59, *p* = 0.55	*β* = − 0.26,t = − 1.18, *p* = 0.24	*β* = 0.18,t = 1.35, *p* = 0.18	*β* = − 0.28,t = − 1.31, *p* = 0.19
Parent routine	*β* = − 0.03,t = − 0.19, *p* = 0.85	*β* = − 0.17,t = − 1.67, *p* = 0.1	*β* = 0.04,t = 0.24, *p* = 0.81	*β* = − 0.02,t = − 0.19, *p* = 0.85	*β* = − 0.08,t = − 0.36, *p* = 0.72	*β* = 0.28,t = 2.19, *p* = 0.03	*β* = − 0.31,t = − 1.46, *p* = 0.14

*p < .05. **p < .01. ***p < .001.

## Discussion

In this study, we examined anxiety in individuals with WS and DS across the world using multilevel modelling with control factors age and gender and the country of residence as well as family identification code as random factors. Concerns, ER frequency and efficacy were also studied to gain a better insight in possible elements that could lead to potentially higher levels of anxiety in individuals with WS and DS.

As predicted, individuals with WS experienced heightened anxiety levels compared to individuals with DS across the three time-points. Taking a closer look, anxiety levels were only higher between the first and second timepoint and the first and third timepoint for both groups. But no difference between the second and third time-point. Our findings align with previous evidence reporting that those with DS experience attenuated psychiatric levels of mental health in general^33^ and during the COVID-19 pandemic when compared with young people with other NDCs or typically developing populations in the UK^45^ and in China^41^ in the early lockdowns. The current work not only provides evidence that those with WS experience elevated anxiety, as researchers previously argued^4,19,26^, but it also presents similar findings in a global setting during a time of crisis, the COVID-19 pandemic. Such findings are important as they help us understand better the mental health differences between NDC populations, but also how they could potentially respond to other similar uncertain crises which induce feelings of anxiety and stress (e.g., school transition, moving into a new house) (see limitations section for discussion of anxiety measure).

Existing research suggested that those with WS also experience more mental health difficulties as they get older^26^. When looking at the controlled factors, our findings show that the older individuals with WS were the higher their anxiety scores were. Whilst it has previously been reported that gender plays a key role in mental health (in the typical developing population) with females being twice as likely to have anxiety^58^, our study suggests that this is not the case in the current NDC populations as there were no significant gender differences (see limitations section for further discussion on gender). Considering the global scale of our data and the number of participants, this is an important finding as it stresses that mental health in individuals with WS and DS was not gender driven during the COVID-19 pandemic and could potentially be routed in other factors that need to be explored (individual; family or even social level factors such as health problems or family conflict as researchers showed in the UK population^23^).

For our second hypothesis, we predicted individuals with WS to be more concerned compared to those with DS due to their increased anxiety levels and clinical profiles compared to DS^4,19,26^. Indeed, those with WS were consistently more concerned about health- and social-related concerns and for some items in the school-related concerns. Previous research has highlighted the fact that those with WS are often more concerned about health^26^. The present study is the first one to report that individuals with WS were concerned about their health during the COVID-19 pandemic. It also replicates previous findings which argue that those with DS scored lower for health-related concerns during the third lockdown in the UK^45^.

To begin with, researchers discussed how those with NDCs were more concerned about their social aspects of their lives than typically developing individuals^68^. Others though argued that both young people with NDCs and typically developing ones showed similar levels of social-related concerns in the UK^23^. Our results do not only show that individuals with WS were more concerned about social-related concerns compared to those with DS, but these findings replicated in a global setting. Another possible explanation for the high levels of social-related concerns of individuals with WS could be the fact that this population is highly sociable, and those with WS enjoy being accompanied by others during challenging and difficult times^16^. During the pandemic, individuals with WS missed such interactions due to several restrictions such as school-closures and shielding which could have possibly led to elevated concerns about social aspects.

Loss of institutional support was a concern for both individuals with WS and DS to a similar extent. During the COVID-19 pandemic, all individuals with NDCs (inclusive of WS and DS) had to abstain from school due to shielding during the pandemic^59^; hence leading to a loss of institutional support. In terms of concerns around boredom and loss of routine, there were key differences with those with DS scoring lower. Our results are consistent with prior research conducted in the UK samples^45^ where individuals with DS scored lower in various areas, such as concerns related to family context. Additionally, both individuals with WS and DS also scored low in financial concerns which could be explained by the limited knowledge these individuals have about financial concepts and their struggles with mathematical concepts as noted in earlier studies^69,70^.

When accounting for the fixed factors, our results showed that one’s gender had no bearing on the measured concerns. Further research is needed to understand the effect of gender on concerns and anxiety in the NDC populations. As for age, those who were older were more concerned about several aspects of their lives. Of course, it is anticipated that the older the individual the better the understanding of the situations^71^, even in the atypical population.

Finally, we hypothesised that individuals with WS will engage more frequently in some (maladaptive) ER strategies considering their heightened levels of anxiety^4^ and maladaptive behaviours^31^. As it was assumed, individuals with WS engaged more in ER strategies such as searching for more information, rumination, repetitive behaviours, and sharing/talking about COVID-19 and focusing on the positive. Some of these strategies might indeed have an alleviating effect on anxiety, and some could be unsuccessful when it comes to reducing anxiety levels^50,51^. When accounting for the control factors, gender did not play a key role (see limitations section for further discussion). This contrasts with the typically developing literature where women are reported to use more ER strategies than men do, especially as they tend to engage more in rumination^72^. Nevertheless, the ER literature is lacking vital information on how men regulate their emotions^72^. Overall, there was an increase in engagement and frequency of ER strategies by the older participants which aligns with the literature that the older the individual the more motivated to engage more frequently in ER to regulate negative affect^73^. Our findings justify such evidence as individuals with DS engaged more with ER strategy humour compared to individuals with WS who engaged less as the interaction of the model revealed. A possible explanation to this could be the fact that individuals with DS have a better understanding of humour due to their less impaired cognitive skills compared to individuals with WS^9,10^.

When looking at the efficacy of the ER strategies, no differences between individuals with DS and WS were revealed. These findings are unique as they provide new evidence about the ER efficacy in periods of crisis for both individuals with WS and DS. Nonetheless, these outcomes should be replicated first in both times of crisis and non-crisis as the literature on frequency and efficacy of ER strategies is limited and possible comparisons on ER frequency and efficacy could reveal interesting outcomes. As for the control factors, only gender played a significant role. Gender was found to impact on isolation/withdrawal, information avoidance, and sharing/talking about COVID-19 and distraction with more women efficiently using these strategies. Research from the literature of Autism, suggests that both gender and age can play a key role in ER strategies and their efficacy ^54^. However, the current study only revealed gender differences. Overall, the current work adds new evidence to the field of ER for both frequency and efficacy for individuals with WS and DS as the literature for these populations is limited.

### Conclusions

To our knowledge, this is the first study to investigate an international perspective on levels of anxiety, concerns, and frequency as well as efficacy of ER strategies in individuals with WS and DS during the COVID-19 outbreak. The findings highlight that there are key differences in anxiety over time between WS and DS, with individuals with WS showing higher anxiety and concerns, especially for those who were older. These high levels of anxiety are possibly triggered by a broader range of concerns and ER strategies that individuals with WS seemed to engage more with, compared to those with DS. For instance, it appears that those with DS not only experienced lower levels of anxiety, but they also engaged less in information search and rumination ER strategies. In addition, those with DS seemed to be less concerned about social-related aspects. Regardless of all the differences, both those with WS and DS experienced lower concerns regarding loss of institutional support and family-related matters. In terms of ER strategies, it is possible that lower anxiety may be a result of adaptive strategy use or the fact that lower anxiety allows individuals to use such strategies more to cope. At present, it is difficult to draw any conclusions due to the limited research available on ER in both WS and DS populations.

Overall, to disentangle this complex interplay, we need to further research on ER and anxiety in longitudinal designs or in studies that ask for the habitual use of participants, not just only assessing what they are doing “right now” to reduce their anxiety levels.

### Limitations and future studies

Although these data are originated from the early days of the COVID-19 pandemic, they provide great insights into profiles of anxiety, concerns, and ER patterns in individuals with WS and DS. While we have a large sample size, the sample is not characterized very well. For example, we have more data from some countries than from others, hence our sample can be considered skewed (see supplementary materials for more information). Still, this is the first time that such a large dataset has been obtained for individuals with WS and DS. In addition, due to the fast response to the pandemic, there are many single item scales in the data (e.g., anxiety, concerns) which do not capture the construct and have less sensitivity as well as reliability. However, recent research evidence argues that humans can rate their feelings accurately using an integer scale^74^ which could explain why our results do overlap with findings from previous studies that have used standardised measures of mental health. Furthermore, it is important to take into consideration that these data were parent reported. Previous research suggests that parents are good at estimating their children’s mental health and behavioural problems, but recent evidence states otherwise^75^. Hence it is possible that parents misreported demographic (e.g., age) and mental health data related to their child.

Therefore, moving forwards, it is vital to employ standardised methods of measurement to ensure that the constructs are measured reliably. Furthermore, it will be important to understand better the differences in ER strategies between individuals with WS and DS through longitudinal designs which measure the impact of other life crises throughout their development such as school-transitions and how these changes impact them.

## Supplementary Information

Supplementary Information 1.

Supplementary Information 2.

Supplementary Information 3.

Supplementary Information 4.

Supplementary Information 5.

## Data Availability

The questionnaire and data will be fully available on OSF (https://osf.io/5nkq9/) and from the corresponding author upon reasonable request.

## Code availability

The code will be available on OSF (https://osf.io/5nkq9/) and from corresponding author upon request.
