# Supplementary material for: Anxiety, concerns and emotion regulation in individuals with Williams syndrome and Down syndrome during the COVID-19 outbreak: a global study
Source: Sci Rep. 2023 May 20;13:8177. doi: 10.1038/s41598-023-35176-7 (PMC10199450; doi:10.1038/s41598-023-35176-7)
Supplement: Supplementary file 1 — Supplementary Information 1. [file 41598_2023_35176_MOESM1_ESM.docx]

1. **Frequency per country**

***Frequency of residency in the countries ordered from highest to lowest.***

| **Country** | **Down** **Syndrome** | **Williams Syndrome** | **Total** |
| --- | --- | --- | --- |
| Saudi Arabia | 163 | 3 | 166 |
| Germany | 84 | 60 | 144 |
| United Kingdom | 68 | 30 | 98 |
| United States | 21 | 68 | 89 |
| Switzerland | 65 | 12 | 77 |
| Netherlands | 29 | 1 | 30 |
| Belgium | 25 | 0 | 25 |
| Spain | 16 | 7 | 23 |
| France | 8 | 14 | 22 |
| Italy | 5 | 16 | 21 |
| China | 17 | 0 | 17 |
| Russia | 8 | 7 | 15 |
| Slovakia | 1 | 13 | 14 |
| Czech Republic | 5 | 6 | 11 |
| Ireland | 3 | 5 | 8 |
| Austria | 6 | 0 | 6 |
| Portugal | 6 | 0 | 6 |
| Sweden | 5 | 1 | 6 |
| New Zealand | 5 | 0 | 5 |
| Canada | 1 | 2 | 3 |
| Luxembourg | 3 | 0 | 3 |
| Australia | 2 | 0 | 2 |
| Iran | 2 | 0 | 2 |
| Romania | 2 | 0 | 2 |
| Brazil | 1 | 0 | 1 |
| Ecuador | 1 | 0 | 1 |
| Greece | 0 | 1 | 1 |
| India | 1 | 0 | 1 |
| Norway | 0 | 1 | 1 |
| Peru | 1 | 0 | 1 |
| South Africa | 1 | 0 | 1 |
| Swaziland | 1 | 0 | 1 |
| Tanzania | 1 | 0 | 1 |

*Note. N =* 804*.*
