# Supplementary material for: Anxiety, concerns and emotion regulation in individuals with Williams syndrome and Down syndrome during the COVID-19 outbreak: a global study
Source: Sci Rep. 2023 May 20;13:8177. doi: 10.1038/s41598-023-35176-7 (PMC10199450; doi:10.1038/s41598-023-35176-7)
Supplement: Supplementary file 2 — Supplementary Information 2. [file 41598_2023_35176_MOESM2_ESM.docx]

**Supplementary Materials B – Generalised Linear Mixed Models for Anxiety, Concerns, ER Frequency and ER Efficacy.**

**Outputs for Anxiety Model.**

**Main effects:**

**Age:** χ2(1) = 17.43, p = .000

**Gender:** χ2(1) = .02, p = .86

**Diagnosis:** χ2(1) = 85.39, p = .000

**Time:** χ2(2) = 49.10, p = .000

**Diagnosis: Time:** χ2(2) = 1.33, p = .51

**Post-hoc statistics**

**Diagnosis Contrasts:**

**Before the pandemic; DS – WS:** β = -.38, z=-6.50, p < .0001

**At the beginning of the pandemic; DS - WS:** β = -.38, z=-7.05, p < .0001

**Now; DS - WS:** β = -.31, z=-5.8, p = .0001

**Time Contrasts:**

**DS:**

**Before the pandemic – At the beginning of the pandemic:** β = -.17, z=-3.91, p = .0003

**Before the pandemic – Now:** β = -.25, z=-5.89, p < .0001

**At the beginning of the pandemic – Now:** β = -.08, z=-2.0, p = .112

**WS:**

**Before the pandemic – At the beginning of the pandemic:** β = -.18, z=-3.27, p = .0030

**Before the pandemic – Now:** β = -.19, z=-3.54, p = .0011

**At the beginning of the pandemic – Now:** β = -.01, z=-.27, p = .96

**Outputs for Concerns.**

**Table 1:** *Effects of group, age, and gender within the multilevel analyses on concerns.*

| **Concerns** |  | **Grou *p*** | **Age** | **Age*Grou *p*** | **Gender** | **Gender*Grou *p*** | **Gender*Age** | **Gender*Age*Grou *p*** |
| --- | --- | --- | --- | --- | --- | --- | --- | --- |
| **Illness in general** |  | χ2(1) = 31.56,p = 1.93e-08 | χ2(1) = 35.35,p = 2.76e-09 | χ2(1) = 0.48,p = 0.49 | χ2(1) = 0.81,p = 0.37 | χ2(1) = 1.34,p = 0.25 | χ2(1) = 0.41,p = 0.52 | χ2(1) = 1.34,p = 0.25 |
| **COVID-19 general** |  | χ2(1) = 24.63,p = 6.94e-07 | χ2(1) = 50.99,p = 9.30e-13 | χ2(1) = 2.49,p = 0.11 | χ2(1) = 0.68,p = 0.41 | χ2(1) = 2.6,p = 0.11 | χ2(1) = 0.16,p = 0.69 | χ2(1) = 0.25,p = 0.62 |
| **Family’s safe ty** |  | χ2(1) = 21.82,p = 2.99e-06 | χ2(1) = 37.41,p = 9.54e-10 | χ2(1) = 1.22,p = 0.27 | χ2(1) = 0.42,p = 0.52 | χ2(1) = 2.02,p = 0.16 | χ2(1) = 0.31,p = 0.58 | χ2(1) = 0.1,p = 0.75 |
| **Own heal th** |  | χ2(1) = 37.89,p = 7.46e-10 | χ2(1) = 34.80,p = 3.65e-09 | χ2(1) = 1.08,p = 0.30 | χ2(1) = 0.53,p = 0.47 | χ2(1) = 1.36,p = 0.24 | χ2(1) = 0.18,p = 0.67 | χ2(1) = 1.19,p = 0.28 |
| **Loss of social con tac t** |  | χ2(1) = 9.28,p = 0.002 | χ2(1) = 5.07,p = 0.02 | χ2(1) = 3.83,p = 0.05 | χ2(1) = 0.44,p = 0.51 | χ2(1) = 0.05,p = 0.83 | χ2(1) = 0.09,p = 0.77 | χ2(1) = 0.04,p = 0.84 |
| **No t able to a p proach o thers** |  | χ2(1) = 19.3,p = 1.12e-05 | χ2(1) = 11.07,p = 0.0008 | χ2(1) = 5.34,p = 0.02 | χ2(1) = 0.27,p = 0.61 | χ2(1) = 0.01,p = 0.91 | χ2(1) = 0.41,p = 0.52 | χ2(1) = 0.01,p = 0.94 |
| **Loss of rou tine** |  | χ2(1) = 4.24,p = 0.04 | χ2(1) = 4.82,p = 0.03 | χ2(1) = 0.16,p = 0.69 | χ2(1) = 0.16,p = 0.69 | χ2(1) = 0.08,p = 0.77 | χ2(1) = 0.65,p = 0.42 | χ2(1) = 0.46,p = 0.5 |
| **Boredom** |  | χ2(1) = 6.36,p = 0.01 | χ2(1) = 4.19,p = 0.04 | χ2(1) = 0.04,p = 0.83 | χ2(1) = 0.01,p = 0.92 | χ2(1) = 0.08,p = 0.78 | χ2(1) = 0.02,p = 0.89 | χ2(1) = 0.06,p = 0.8 |
| **COVID-19 self** |  | χ2(1) = 28.26,p = 1.06e-07 | χ2(1) = 39.64,p = 3.05e-10 | χ2(1) = 0.36,p = 0.55 | χ2(1) = 0.08,p = 0.78 | χ2(1) = 1.97,p = 0.16 | χ2(1) = 0.35,p = 0.56 | χ2(1) = 1.32,p = 0.25 |
| **COVID-19 o thers** |  | χ2(1) = 29.35,p = 6.05e-08 | χ2(1) = 39.38,p = 3.49e-10 | χ2(1) = 5.16,p = 0.02 | χ2(1) = 0.06,p = 0.81 | χ2(1) = 5.66,p = 0.02 | χ2(1) = 0.79,p = 0.37 | χ2(1) = 0.2,p = 0.66 |
| **Loss of ins ti tu tional su p por t** |  | χ2(1) = 0.13,p = 0.72 | χ2(1) = 0.41,p = 0.52 | χ2(1) = 0.01,p = 0.93 | χ2(1) = 0.41,p = 0.52 | χ2(1) = 0.03,p = 0.86 | χ2(1) = 1.31,p = 0.25 | χ2(1) = 0.01,p = 0.92 |
| **Family conflic t** |  | χ2(1) = 2.07,p = 0.15 | χ2(1) = 0.13,p = 0.72 | χ2(1) = 0.72,p = 0.39 | χ2(1) = 0.2,p = 0.66 | χ2(1) = 0.19,p = 0.66 | χ2(1) = 0.93,p = 0.34 | χ2(1) = 0.82,p = 0.36 |
| **Financial concerns** |  | χ2(1) = 0.81,p = 0.37 | χ2(1) = 3.03,p = 0.08 | χ2(1) = 0.51,p = 0.47 | χ2(1) = 2.36,p = 0.12 | χ2(1) = 1.01,p = 0.32 | χ2(1) = 0.1,p = 0.75 | χ2(1) = 0,p = 0.98 |

**Table 2:** *Estimates of group, age, and gender within the multilevel analyses on concerns.*

| **Concerns** |  | **Grou *p*** | **Age** | **Age*Grou *p*** | **Gender** | **Gender*Grou *p*** | **Gender*Age** | **Gender*Age*Grou *p*** |
| --- | --- | --- | --- | --- | --- | --- | --- | --- |
| **Illness in general** |  | β =0.37,z =4.83,p = 1.38e-05 | β =0.20,z =4.06,p = 4.96e-05 | β =-0.1,z =-1.33,p = 0.18 | β =0.01,z =0.15,p = 0.88 | β =-0.14,z =-1.34,p = 0.18 | β =-0.08,z =-1.24,p = 0.21 | β =0.11,z =1.16,p = 0.25 |
| **COVID-19 general** |  | β =0.37,z =4.77,p = 1.82e-06 | β =0.21,z =4.46,p = 8.18e-06 | β =-0.1,z =-1.41,p = 0.16 | β =0.03,z =0.44,p = 0.66 | β =-0.18,z =-1.68,p = 0.09 | β =-0.04,z =-0.63,p = 0.53 | β =0.05,z =0.5,p = 0.62 |
| **Family’s safe ty** |  | β =0.34,z =4.34,p = 1.44e-05 | β =0.19,z =3.79,p = 0.000 | β =-0.07,z =-0.97,p = 0.33 | β =0.03,z =0.44,p = 0.66 | β =-0.16,z =-1.46,p = 0.15 | β =-0.04,z =-0.63,p = 0.53 | β =0.03,z =0.32,p = 0.75 |
| **Own heal th** |  | β =0.4,z =5.21,p = 1.84e-07 | β =0.20,z =4.00,p = 6.33e-05 | β =-0.11,z =-1.5,p = 0.13 | β =0.02,z =0.27,p = 0.79 | β =-0.14,z =-1.33,p = 0.18 | β =-0.07,z =-1.04,p = 0.3 | β =0.1,z =1.09,p = 0.28 |
| **Loss of social con tac t** |  | β =0.16,z =2.23,p = 0.03 | β =0.09,z =2.13,p = 0.03 | β =-0.09,z =-1.41,p = 0.16 | β =-0.03,z =-0.65,p = 0.51 | β =0.02,z =0.2,p = 0.84 | β =-0.02,z =-0.36,p = 0.72 | β =0.02,z =0.2,p = 0.84 |
| **No t able to a p proach o thers** |  | β =0.24,z =3.32,p = 0.0008 | β =0.13,z =2.87,p = 0.004 | β =-0.10,z =-1.44,p = 0.15 | β =-0.02,z =-0.44,p = 0.66 | β =0.01,z =0.12,p = 0.91 | β =-0.02,z =-0.45,p = 0.66 | β =-0.01,z =-0.08,p = 0.94 |
| **Loss of rou tine** |  | β =0.1,z =1.3,p = 0.19 | β =0.06,z =1.34,p = 0.18 | β =0.02,z =0.26,p = 0.8 | β =0.01,z =0.19,p = 0.85 | β =0.03,z =0.35,p = 0.73 | β =-0.01,z =-0.19,p = 0.85 | β =-0.06,z =-0.68,p = 0.5 |
| **Boredom** |  | β =0.11,z =1.5,p = 0.13 | β =0.06,z =1.27,p = 0.2 | β =-0.02,z =-0.33,p = 0.74 | β =-0.01,z =-0.25,p = 0.8 | β =0.03,z =0.25,p = 0.8 | β =-0.02,z =-0.27,p = 0.79 | β =0.02,z =0.25,p = 0.8 |
| **COVID-19 self** |  | β =0.38,z =4.75,p = 2.05e-06 | β =0.21,z =4.09,p = 4.35e-05 | β =-0.1,z =-1.27,p = 0.21 | β =0.08,z =1.22,p = 0.22 | β =-0.17,z =-1.59,p = 0.11 | β =-0.08,z =-1.2,p = 0.23 | β =0.11,z =1.15,p = 0.25 |
| **COVID-19 o thers** |  | β =0.45,z =5.71,p = 1.15e-08 | β =0.22,z =4.52,p = 6.24e-06 | β =-0.13,z =-1.8,p = 0.07 | β =0.12,z =1.79,p = 0.07 | β =-0.25,z =-2.42,p = 0.02 | β =-0.06,z =-0.97,p = 0.33 | β =0.04,z =0.45,p = 0.66 |
| **Loss of ins ti tu tional su p por t** |  | β =0.03,z =0.38,p = 0.71 | β =0.05,z =1.01,p = 0.31 | β =0,z =-0.03,p = 0.98 | β =0.04,z =0.61,p = 0.54 | β =-0.02,z =-0.19,p = 0.85 | β =-0.06,z =-0.96,p = 0.34 | β =0.01,z =0.11,p = 0.92 |
| **Family conflic t** |  | β =0.12,z =1.32,p = 0.19 | β =0.03,z =0.6,p = 0.55 | β =0.01,z =0.13,p = 0.89 | β =0.04,z =0.65,p = 0.51 | β =-0.05,z =-0.39,p = 0.69 | β =-0.01,z =-0.19,p = 0.85 | β =-0.1,z =-0.91,p = 0.36 |
| **Financial concerns** |  | β =0.14,z =1.26,p = 0.21 | β =0.05,z =0.74,p = 0.46 | β =0.05,z =0.47,p = 0.64 | β =0.14,z =1.84,p = 0.07 | β =-0.14,z =-0.99,p = 0.32 | β =-0.02,z =-0.24,p = 0.81 | β =0,z =-0.02,p = 0.98 |

**Outputs for ER Frequency.**

**Table 3:** *Effects of group, age, and gender within the multilevel analyses on emotion regulation (ER) frequency.*

| **ER Strategies** | **Group** | **Age** | **Age*Group** | **Gender** | **Gender*Group** | **Gender*Age** | **Gender*Age*Group** |  |
| --- | --- | --- | --- | --- | --- | --- | --- | --- |
| ***Isolation/Withdrawal*** | χ2(1) = 0.46,p = 0.5 | χ2(1) = 22.79,p = 1.81e-06 | χ2(1) = 0.51,p = 0.47 | χ2(1) = 2.85,p = 0.09 | χ2(1) = 0.48,p = 0.49 | χ2(1) = 0.34,p = 0.56 | χ2(1) = 1.64,p = 0.2 |  |
| ***Information Avoidance*** | χ2(1) = 0.03,p = 0.87 | χ2(1) = 13.17,p = 0.0003 | χ2(1) = 0.54,p = 0.46 | χ2(1) = 1.42,p = 0.23 | χ2(1) = 0.16,p = 0.69 | χ2(1) = 3.32,p = 0.07 | χ2(1) = 0.01,p = 0.93 |  |
| ***Information Search*** | χ2(1) = 20.75,p = 5.23e-06 | χ2(1) = 6.11,p = 0.01 | χ2(1) = 1.9,p = 0.17 | χ2(1) = 0.29,p = 0.59 | χ2(1) = 0.77,p = 0.38 | χ2(1) = 0.22,p = 0.64 | χ2(1) = 0.27,p = 0.6 |  |
| ***Rumination*** | χ2(1) = 18.58,p = 1.63e-05 | χ2(1) = 17.17,p = 3.42e-05 | χ2(1) = 1.09,p = 0.30 | χ2(1) = 0.4,p = 0.53 | χ2(1) = 0.35,p = 0.56 | χ2(1) = 0.08,p = 0.78 | χ2(1) = 0.66,p = 0.41 |  |
| ***Expressive suppression*** | χ2(1) = 0.09,p = 0.77 | χ2(1) = 6.43,p = 0.01 | χ2(1) = 1.01,p = 0.31 | χ2(1) = 0.02,p = 0.89 | χ2(1) = 0.32,p = 0.57 | χ2(1) = 0.11,p = 0.74 | χ2(1) = 1.78,p = 0.18 |  |
| ***Aggressive Behaviours*** | χ2(1) = 0.74,p = 0.39 | χ2(1) = 11.07,p = 0.0009 | χ2(1) = 0.05,p = 0.82 | χ2(1) = 2.81,p = 0.09 | χ2(1) = 0.09,p = 0.76 | χ2(1) = 0.52,p = 0.47 | χ2(1) = 2.69,p = 0.1 |  |
| ***Repetitive Behaviours*** | χ2(1) = 34.1,p = 5.23e-09 | χ2(1) = 1,p = 0.32 | χ2(1) = 3.05,p = 0.08 | χ2(1) = 4.42,p = 0.04 | χ2(1) = 0.78,p = 0.38 | χ2(1) = 1.69,p = 0.19 | χ2(1) = 0.29,p = 0.59 |  |
| ***Sharing/Talking about COVID-19*** | χ2(1) = 26.68,p = 2.40e-07 | χ2(1) = 1.86,p = 0.17 | χ2(1) = 4.56,p = 0.03 | χ2(1) = 0.66,p = 0.42 | χ2(1) = 0.69,p = 0.41 | χ2(1) = 0.21,p = 0.65 | χ2(1) = 0,p = 0.99 |  |
| ***Distraction*** | χ2(1) = 4.85,p = 0.03 | χ2(1) = 7.7,p = 0.01 | χ2(1) = 0.28,p = 0.60 | χ2(1) = 1.8,p = 0.18 | χ2(1) = 1.37,p = 0.24 | χ2(1) = 1.99,p = 0.16 | χ2(1) = 0.14,p = 0.71 |  |
| ***Cognitive Reappraisal*** | χ2(1) = 2.35,p = 0.13 | χ2(1) = 7.71,p = 0.01 | χ2(1) = 0.66,p = 0.41 | χ2(1) = 0.73,p = 0.39 | χ2(1) = 0.21,p = 0.65 | χ2(1) = 2.69,p = 0.1 | χ2(1) = 0.13,p = 0.72 |  |
| ***Focusing on the Positive*** | χ2(1) = 6.56,p = 0.01 | χ2(1) = 4.29,p = 0.04 | χ2(1) = 1.54,p = 0.22 | χ2(1) = 0.63,p = 0.43 | χ2(1) = 0.02,p = 0.89 | χ2(1) = 4.1,p = 0.04 | χ2(1) = 0.04,p = 0.85 |  |
| ***Humour*** | χ2(1) = 4.09,p = 0.04 | χ2(1) = 1.03,p = 0.31 | χ2(1) = 5.28,p = 0.02 | χ2(1) = 1.71,p = 0.19 | χ2(1) = 0.73,p = 0.39 | χ2(1) = 1.61,p = 0.2 | χ2(1) = 0.01,p = 0.91 |  |
| ***Parent Shielding*** | χ2(1) = 4.27,p = 0.04 | χ2(1) = 0.12,p = 0.73 | χ2(1) = 0.05,p = 0.82 | χ2(1) = 0.01,p = 0.93 | χ2(1) = 2.08,p = 0.15 | χ2(1) = 0.05,p = 0.83 | χ2(1) = 0.92,p = 0.34 |  |
| ***Parent Routine*** | χ2(1) = 0.01,p = 0.93 | χ2(1) = 0.41,p = 0.52 | χ2(1) = 1.64,p = 0.2 | χ2(1) = 3.11,p = 0.08 | χ2(1) = 0.01,p = 0.94 | χ2(1) = 0.09,p = 0.77 | χ2(1) = 0.1,p = 0.75 |  |

**Table 4:** *Estimates of group, age, and gender within the multilevel analyses on emotion regulation (ER) frequency.*

| **ER Strategies** | **Group** | **Age** | **Age*Group** | **Gender** | **Gender*Group** | **Gender*Age** | **Gender*Age*Group** |  |
| --- | --- | --- | --- | --- | --- | --- | --- | --- |
| ***Isolation/Withdrawal*** | β =-0.07,z =-0.83,p = 0.41 | β =0.13,z =2.92,p = 0.003 | β =-0.11,z =-1.44,p = 0.15 | β =-0.11,z =-1.8,p = 0.07 | β =0.06,z =0.5,p = 0.61 | β =-0.02,z =-0.27,p = 0.78 | β =0.13,z =1.28,p = 0.2 |  |
| ***Information Avoidance*** | β =-0.01,z =-0.10,p = 0.92 | β =0.16,z =3.27,p = 0.0011 | β =-0.04,z =-0.56,p = 0.58 | β =-0.07,z =-1.07,p = 0.28 | β =0.04,z =0.38,p = 0.7 | β =-0.1,z =-1.51,p = 0.13 | β =0.01,z =0.09,p = 0.93 |  |
| ***Information Search*** | β =0.21,z =2.43,p = 0.01 | β =0.12,z =2.26,p = 0.02 | β =-0.11,z =-1.27,p = 0.2 | β =-0.01,z =-0.08,p = 0.94 | β =0.09,z =0.83,p = 0.41 | β =-0.05,z =-0.7,p = 0.49 | β =0.06,z =0.52,p = 0.6 |  |
| ***Rumination*** | β =0.31,z =3.57,p = 0.0003 | β =0.16,z =2.93,p = 0.003 | β =-0.1,z =-1.29,p = 0.2 | β =0.06,z =0.91,p = 0.37 | β =-0.08,z =-0.69,p = 0.49 | β =-0.05,z =-0.74,p = 0.46 | β =0.08,z =0.82,p = 0.41 |  |
| ***Expressive suppression*** | β =0.05,z =0.58,p = 0.56 | β =0.09,z =1.71,p = 0.09 | β =-0.03,z =-0.38,p = 0.7 | β =0.03,z =0.43,p = 0.67 | β =-0.09,z =-0.74,p = 0.46 | β =-0.07,z =-1.08,p = 0.28 | β =0.15,z =1.33,p = 0.18 |  |
| ***Aggressive Behaviours*** | β =0.03,z =0.37,p = 0.71 | β =-0.11,z =-1.87,p = 0.06 | β =0.1,z =1.11,p = 0.27 | β =0.09,z =1.31,p = 0.19 | β =0.03,z =0.24,p = 0.81 | β =0.03,z =0.45,p = 0.65 | β =-0.2,z =-1.64,p = 0.1 |  |
| ***Repetitive Behaviours*** | β =0.24,z =3.09,p = 0.002 | β =0.04,z =0.70,p = 0.48 | β =-0.05,z =-0.68,p = 0.5 | β =0.07,z =1.13,p = 0.26 | β =0.09,z =0.89,p = 0.37 | β =-0.04,z =-0.65,p = 0.51 | β =-0.05,z =-0.54,p = 0.59 |  |
| ***Sharing/Talking about COVID-19*** | β =0.25,z =3.06,p = 0.002 | β =0.06,z =1.22,p = 0.22 | β =-0.11,z =-1.37,p = 0.17 | β =-0.08,z =-1.16,p = 0.25 | β =0.09,z =0.83,p = 0.41 | β =0.02,z =0.34,p = 0.73 | β =0,z =0.02,p = 0.99 |  |
| ***Distraction*** | β =0.06,z =0.77,p = 0.44 | β =0.1,z =2.24,p = 0.03 | β =0,z =-0.07,p = 0.95 | β =-0.1,z =-1.7,p = 0.09 | β =0.12,z =1.2,p = 0.23 | β =-0.05,z =-0.86,p = 0.39 | β =-0.04,z =-0.37,p = 0.71 |  |
| ***Cognitive Reappraisal*** | β =0.08,z =0.84,p = 0.4 | β =0.15,z =2.87,p = 0.004 | β =-0.07,z =-0.81,p = 0.42 | β =-0.06,z =-0.86,p = 0.39 | β =0.05,z =0.41,p = 0.68 | β =-0.11,z =-1.51,p = 0.13 | β =0.04,z =0.36,p = 0.72 |  |
| ***Focusing on the Positive*** | β =0.13,z =1.7,p = 0.09 | β =0.13,z =2.8,p = 0.005 | β =-0.07,z =-0.97,p = 0.33 | β =-0.04,z =-0.64,p = 0.52 | β =0.01,z =0.12,p = 0.9 | β =-0.11,z =-1.71,p = 0.09 | β =0.02,z =0.19,p = 0.85 |  |
| ***Humour*** | β =0.07,z =0.85,p = 0.4 | β =0.11,z =2.18,p = 0.03 | β =-0.13,z =-1.53,p = 0.13 | β =0.04,z =0.59,p = 0.55 | β =0.09,z =0.85,p = 0.4 | β =-0.07,z =-1.08,p = 0.28 | β =0.01,z =0.11,p = 0.91 |  |
| ***Parent Shielding*** | β =0.19,z =2.49,p = 0.01 | β =-0.03,z =-0.55,p = 0.58 | β =0.06,z =0.88,p = 0.38 | β =0.06,z =0.98,p = 0.33 | β =-0.13,z =-1.39,p = 0.17 | β =0.05,z =0.79,p = 0.43 | β =-0.09,z =-0.96,p = 0.34 |  |
| ***Parent Routine*** | β =0.01,z =0.18,p = 0.86 | β =0.01,z =0.18,p = 0.86 | β =-0.04,z =-0.55,p = 0.58 | β =0.08,z =1.48,p = 0.14 | β =-0.01,z =-0.06,p = 0.95 | β =0,z =-0.03,p = 0.97 | β =-0.03,z =-0.32,p = 0.75 |  |

**Outputs for ER efficacy.**

**Table 5:** *Effects of group, age, and gender within the multilevel analyses on emotion regulation (ER) efficacy.*

| **ER Strategies** | **Group** | **Age** | **Age*Group** | **Gender** | **Gender*Group** | **Gender*Age** | **Gender*Age*Group** |  |
| --- | --- | --- | --- | --- | --- | --- | --- | --- |
| ***Isolation/Withdrawal*** | χ2(1) = 3.61,p = 0.06 | χ2(1) = 4.79,p = 0.03 | χ2(1) = 2.22,p = 0.14 | χ2(1) = 6.23,p = 0.01 | χ2(1) = 0.22,p = 0.64 | χ2(1) = 2,p = 0.16 | χ2(1) = 1.15,p = 0.28 |  |
| ***Information Avoidance*** | χ2(1) = 0.14,p = 0.71 | χ2(1) = 1.95,p = 0.16 | χ2(1) = 1.78,p = 0.18 | χ2(1) = 5.81,p = 0.02 | χ2(1) = 0,p = 0.97 | χ2(1) = 2.73,p = 0.1 | χ2(1) = 0.06,p = 0.81 |  |
| ***Information Search*** | χ2(1) = 1.35,p = 0.25 | χ2(1) = 0.01,p = 0.94 | χ2(1) = 1.91,p = 0.17 | χ2(1) = 2.53,p = 0.11 | χ2(1) = 1.78,p = 0.18 | χ2(1) = 0.14,p = 0.71 | χ2(1) = 0.13,p = 0.72 |  |
| ***Rumination*** | χ2(1) = 0.001,p = 0.97 | χ2(1) = 1.31,p = 0.25 | χ2(1) = 1.1,p = 0.29 | χ2(1) = 1.04,p = 0.31 | χ2(1) = 0.09,p = 0.76 | χ2(1) = 0.2,p = 0.65 | χ2(1) = 0.35,p = 0.55 |  |
| ***Expressive suppression*** | χ2(1) = 0.43,p = 0.51 | χ2(1) = 0.03,p = 0.87 | χ2(1) = 0.48,p = 0.49 | χ2(1) = 0.47,p = 0.49 | χ2(1) = 0,p = 0.96 | χ2(1) = 0.1,p = 0.76 | χ2(1) = 1.05,p = 0.31 |  |
| ***Aggressive Behaviours*** | χ2(1) = 0.04,p = 0.84 | χ2(1) = 0.02,p = 0.9 | χ2(1) = 0.08,p = 0.78 | χ2(1) = 0.77,p = 0.38 | χ2(1) = 0.07,p = 0.79 | χ2(1) = 1.13,p = 0.29 | χ2(1) = 1.61,p = 0.2 |  |
| ***Repetitive Behaviours*** | χ2(1) = 4.01,p = 0.05 | χ2(1) = 0.24,p = 0.63 | χ2(1) = 0.83,p = 0.36 | χ2(1) = 1.33,p = 0.25 | χ2(1) = 0.04,p = 0.84 | χ2(1) = 0.23,p = 0.63 | χ2(1) = 0.48,p = 0.49 |  |
| ***Sharing/Talking about COVID-19*** | χ2(1) = 1.11,p = 0.29 | χ2(1) = 0.17,p = 0.68 | χ2(1) = 0.5,p = 0.48 | χ2(1) = 5.45,p = 0.02 | χ2(1) = 0.01,p = 0.94 | χ2(1) = 0.16,p = 0.69 | χ2(1) = 0.31,p = 0.58 |  |
| ***Distraction*** | χ2(1) = 0.31,p = 0.58 | χ2(1) = 0.78,p = 0.38 | χ2(1) = 2.77,p = 0.1 | χ2(1) = 6.96,p = 0.01 | χ2(1) = 1.01,p = 0.32 | χ2(1) = 0.72,p = 0.4 | χ2(1) = 0.18,p = 0.67 |  |
| ***Cognitive Reappraisal*** | χ2(1) = 0.59,p = 0.44 | χ2(1) = 0.25,p = 0.62 | χ2(1) = 3.01,p = 0.08 | χ2(1) = 1.54,p = 0.22 | χ2(1) = 0.09,p = 0.77 | χ2(1) = 0.93,p = 0.33 | χ2(1) = 0.19,p = 0.66 |  |
| ***Focusing on the Positive*** | χ2(1) = 0.01,p = 0.93 | χ2(1) = 0.10,p = 0.76 | χ2(1) = 2.79,p = 0.09 | χ2(1) = 2.26,p = 0.13 | χ2(1) = 0,p = 0.97 | χ2(1) = 2.26,p = 0.13 | χ2(1) = 0.11,p = 0.75 |  |
| ***Humour*** | χ2(1) = 0.05,p = 0.82 | χ2(1) = 0.86,p = 0.35 | χ2(1) = 3.5,p = 0.06 | χ2(1) = 0,p = 0.95 | χ2(1) = 1.32,p = 0.25 | χ2(1) = 0.2,p = 0.65 | χ2(1) = 0.13,p = 0.72 |  |
| ***Parent Shielding*** | χ2(1) = 1.44,p = 0.23 | χ2(1) = 0.25,p = 0.62 | χ2(1) = 1.18,p = 0.28 | χ2(1) = 1.85,p = 0.17 | χ2(1) = 0.88,p = 0.35 | χ2(1) = 0.32,p = 0.57 | χ2(1) = 1.32,p = 0.25 |  |
| ***Parent Routine*** | χ2(1) = 0.06,p = 0.80 | χ2(1) = 0.42,p = 0.52 | χ2(1) = 1.16,p = 0.28 | χ2(1) = 0.25,p = 0.62 | χ2(1) = 0.07,p = 0.79 | χ2(1) = 1.68,p = 0.19 | χ2(1) = 1.42,p = 0.23 |  |

**Table 6:** *Estimates of group, age, and gender within the multilevel analyses on emotion regulation (ER) efficacy.*

| **ER Strategies** | **Group** | **Age** | **Age*Group** | **Gender** | **Gender*Group** | **Gender*Age** | **Gender*Age*Group** |  |
| --- | --- | --- | --- | --- | --- | --- | --- | --- |
| ***Isolation/Withdrawal*** | β =-0.13,z =-1.62,p = 0.11 | β =0.06,z =1.27,p = 0.2 | β =-0.13,z =-1.78,p = 0.08 | β =-0.13,z =-2.38,p = 0.02 | β =0.04,z =0.38,p = 0.7 | β =0.03,z =0.54,p = 0.59 | β =0.11,z =1.07,p = 0.28 |  |
| ***Information Avoidance*** | β =0.03,z =0.33,p = 0.74 | β =0.1,z =2.09,p = 0.04 | β =-0.05,z =-0.73,p = 0.47 | β =-0.11,z =-1.96,p = 0.05 | β =0.01,z =0.06,p = 0.96 | β =-0.07,z =-1.17,p = 0.24 | β =-0.02,z =-0.24,p = 0.81 |  |
| ***Information Search*** | β =0,z =-0.06,p = 0.96 | β =0.03,z =0.63,p = 0.53 | β =-0.05,z =-0.63,p = 0.53 | β =-0.12,z =-2.06,p = 0.04 | β =0.14,z =1.35,p = 0.18 | β =-0.01,z =-0.08,p = 0.93 | β =-0.04,z =-0.36,p = 0.72 |  |
| ***Rumination*** | β =0.02,z =0.26,p = 0.79 | β =0.07,z =1.52,p = 0.13 | β =-0.09,z =-1.13,p = 0.26 | β =-0.04,z =-0.68,p = 0.5 | β =-0.04,z =-0.34,p = 0.73 | β =-0.04,z =-0.72,p = 0.47 | β =0.06,z =0.59,p = 0.55 |  |
| ***Expressive suppression*** | β =-0.04,z =-0.46,p = 0.65 | β =0.05,z =1,p = 0.32 | β =-0.1,z =-1.23,p = 0.22 | β =-0.04,z =-0.63,p = 0.53 | β =0,z =0.01,p = 1 | β =-0.05,z =-0.85,p = 0.39 | β =0.11,z =1.02,p = 0.31 |  |
| ***Aggressive Behaviours*** | β =-0.03,z =-0.41,p = 0.68 | β =0,z =-0.02,p = 0.98 | β =0.09,z =1.14,p = 0.26 | β =-0.05,z =-0.81,p = 0.42 | β =0.04,z =0.35,p = 0.72 | β =0,z =-0.06,p = 0.95 | β =-0.14,z =-1.27,p = 0.2 |  |
| ***Repetitive Behaviours*** | β =0.12,z =1.45,p = 0.15 | β =0,z =0.05,p = 0.96 | β =0,z =-0.05,p = 0.96 | β =0.05,z =0.84,p = 0.4 | β =0.02,z =0.22,p = 0.83 | β =0,z =0.07,p = 0.94 | β =-0.07,z =-0.69,p = 0.49 |  |
| ***Sharing/Talking about COVID-19*** | β =0.06,z =0.75,p = 0.45 | β =0,z =-0.02,p = 0.98 | β =0,z =-0.05,p = 0.96 | β =-0.11,z =-1.92,p = 0.05 | β =0.01,z =0.1,p = 0.92 | β =0.04,z =0.65,p = 0.51 | β =-0.05,z =-0.56,p = 0.58 |  |
| ***Distraction*** | β =-0.01,z =-0.18,p = 0.86 | β =0.06,z =1.42,p = 0.16 | β =-0.06,z =-0.79,p = 0.43 | β =-0.15,z =-2.71,p = 0.01 | β =0.1,z =1.03,p = 0.3 | β =-0.02,z =-0.42,p = 0.67 | β =-0.04,z =-0.42,p = 0.67 |  |
| ***Cognitive Reappraisal*** | β =0.03,z =0.32,p = 0.75 | β =0.04,z =0.77,p = 0.44 | β =-0.07,z =-0.82,p = 0.41 | β =-0.07,z =-1.19,p = 0.23 | β =0.03,z =0.3,p = 0.76 | β =-0.03,z =-0.51,p = 0.61 | β =-0.05,z =-0.43,p = 0.66 |  |
| ***Focusing on the Positive*** | β =0.01,z =0.09,p = 0.93 | β =0.05,z =1.21,p = 0.23 | β =-0.06,z =-0.88,p = 0.38 | β =-0.07,z =-1.26,p = 0.21 | β =0,z =0.05,p = 0.96 | β =-0.06,z =-1.01,p = 0.31 | β =-0.03,z =-0.32,p = 0.75 |  |
| ***Humour*** | β =-0.05,z =-0.58,p = 0.56 | β =0.02,z =0.33,p = 0.74 | β =-0.07,z =-0.91,p = 0.36 | β =-0.04,z =-0.7,p = 0.49 | β =0.12,z =1.15,p = 0.25 | β =-0.01,z =-0.15,p = 0.88 | β =-0.04,z =-0.36,p = 0.72 |  |
| ***Parent Shielding*** | β =0.11,z =1.59,p = 0.11 | β =-0.03,z =-0.74,p = 0.46 | β =0.01,z =0.17,p = 0.87 | β =-0.03,z =-0.48,p = 0.63 | β =-0.08,z =-0.9,p = 0.37 | β =0.07,z =1.16,p = 0.24 | β =-0.1,z =-1.15,p = 0.25 |  |
| ***Parent Routine*** | β =0.01,z =0.09,p = 0.93 | β =-0.05,z =-1.27,p = 0.2 | β =0.02,z =0.23,p = 0.82 | β =-0.01,z =-0.15,p = 0.88 | β =-0.02,z =-0.24,p = 0.81 | β =0.09,z =1.76,p = 0.08 | β =-0.1,z =-1.19,p = 0.23 |  |
