# Supplementary material for: Anxiety, concerns and emotion regulation in individuals with Williams syndrome and Down syndrome during the COVID-19 outbreak: a global study
Source: Sci Rep. 2023 May 20;13:8177. doi: 10.1038/s41598-023-35176-7 (PMC10199450; doi:10.1038/s41598-023-35176-7)
Supplement: Supplementary file 3 — Supplementary Information 3. [file 41598_2023_35176_MOESM3_ESM.docx]

1. **Concerns frequency per group**

*This table includes the average use of concerns and confidence interval for individuals with WS and DS.*

|  | **WS** | | | **DS** | |  |
| --- | --- | --- | --- | --- | --- | --- |
| Concerns | *M* | 95% CI | *M* | | 95% CI | |
| Loss of social contact | 3.39 | [3.22, 3.39] | 2.64 | | [2.52, 2.76] | |
| Not able to approach others | 3.09 | [2.91, 3.09] | 2.36 | | [2.24, 2.47] | |
| Loss of routine | 2.86 | [2.68, 2.86] | 2.43 | | [2.31, 2.55] | |
| COVID-19 General | 2.46 | [2.29, 2.46] | 1.83 | | [1.73, 1.94] | |
| Own health | 2.47 | [2.3, 2.47] | 1.74 | | [1.64, 1.84] | |
| Illness in general | 2.47 | [2.3, 2.46] | 1.78 | | [1.68, 1.88] | |
| COVID-19 Others | 2.51 | [2.34, 2.51] | 1.83 | | [1.73, 1.94] | |
| Loss of institutional support | 2.45 | [2.27, 2.45] | 2.26 | | [2.14, 2.38] | |
| Boredom | 2.43 | [2.26, 2.43] | 2.12 | | [2.01, 2.23] | |
| COVID-19 self | 2.34 | [2.17, 2.34] | 1.71 | | [1.61, 1.8] | |
| Family’s safety | 2.33 | [2.16, 2.33] | 1.76 | | [1.61, 1.86] | |
| Family conflict | 1.84 | [1.69, 1.84] | 1.7 | | [1.61, 1.8] | |
| Financial concerns | 1.24 | [1.14, 1.24] | 1.27 | | [1.2, 1.34] | |

*Note*. CI = confidence interval; DS = Down syndrome, WS = Williams syndrome.
