# Supplementary figures and images for: Anxiety, concerns and emotion regulation in individuals with Williams syndrome and Down syndrome during the COVID-19 outbreak: a global study
Source: Sci Rep. 2023 May 20;13:8177. doi: 10.1038/s41598-023-35176-7 (PMC10199450; doi:10.1038/s41598-023-35176-7)

1. **Concerns Significant Interactions**


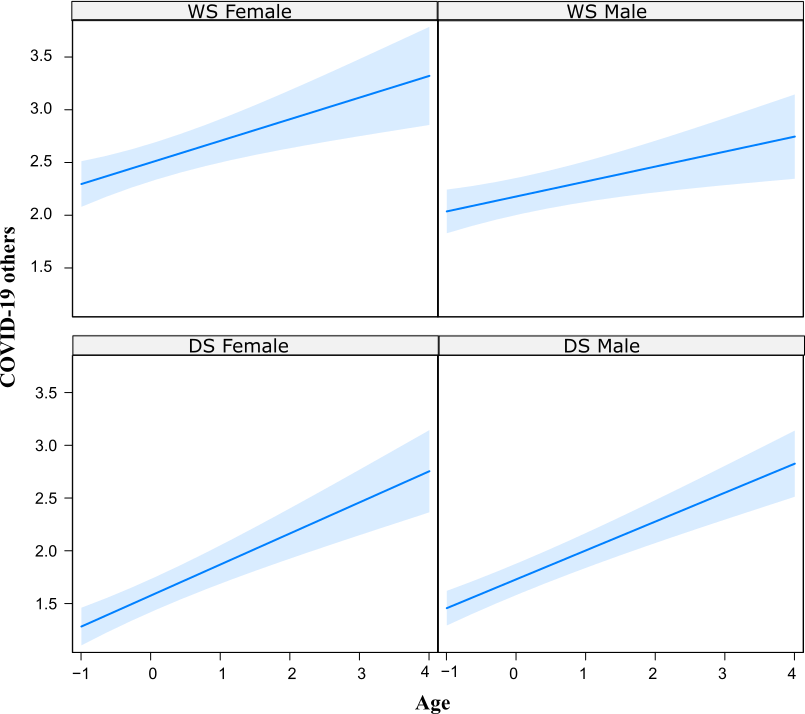

Supplement: Supplementary file 4 — Supplementary Information 4. [file 41598_2023_35176_MOESM4_ESM.docx]

1. **Emotion Regulation Frequency Significant Interaction**


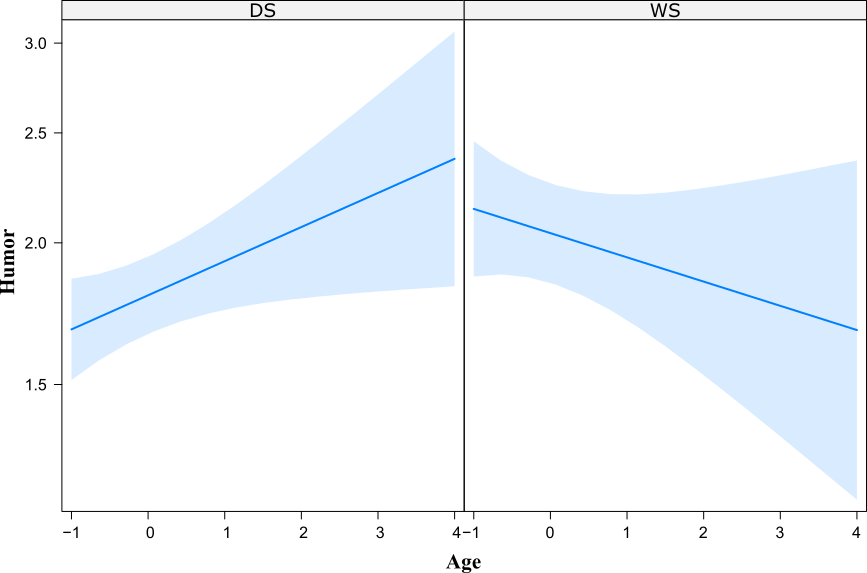

Supplement: Supplementary file 5 — Supplementary Information 5. [file 41598_2023_35176_MOESM5_ESM.docx]
